# Co-exposure effects of urinary polycyclic aromatic hydrocarbons and metals on lung function: mediating role of systematic inflammation

**DOI:** 10.1186/s12890-024-03173-9

**Published:** 2024-08-11

**Authors:** Lihong Wu, Xue Lu, Siying Zhang, Yumei Zhong, Hui Gao, Fang-biao Tao, Xiulong Wu

**Affiliations:** 1 Department of Maternal, Child and Adolescent Health, School of Public Health, Anhui Medical University, No 81 Meishan Road, Hefei, 230032 Anhui China; 2 Key Laboratory of Population Health Across Life Cycle (Anhui Medical University), Ministry of Education of the People’s Republic of China, No 81 Meishan Road, Hefei, 230032 Anhui China; 3 Anhui Provincial Key Laboratory of Environment and Population Health Across the Life Course, Anhui Medical University, No 81 Meishan Road, Hefei, 230032 Anhui China; 4 Department of Toxicology, Anhui Medical University, Anhui, China; 5 Department of Pediatrics, The First Affiliated Hospital of Anhui Medical University, No 218 Jixi Road, Hefei, 230022 Anhui China

**Keywords:** Polycyclic aromatic hydrocarbons, Metals, Lung function, Mixture effect, White blood cell

## Abstract

**Background:**

Polycyclic aromatic hydrocarbons (PAHs) and metals were associated with decreased lung function, but co-exposure effects and underlying mechanism remained unknown.

**Methods:**

Among 1,123 adults from National Health and Nutrition Examination Survey 2011–2012, 10 urinary PAHs, 11 urinary metals, and peripheral white blood cell (WBC) count were determined, and 5 lung function indices were measured. Least absolute shrinkage and selection operator, Bayesian kernel machine regression, and quantile-based g-computation were used to estimate co-exposure effects on lung function. Mediation analysis was used to explore mediating role of WBC.

**Results:**

These models demonstrated that PAHs and metals were significantly associated with lung function impairment. Bayesian kernel machine regression models showed that comparing to all chemicals fixed at median level, forced expiratory volume in 1 s (FEV_1_)/forced vital capacity, peak expiratory flow, and forced expiratory flow between 25 and 75% decreased by 1.31% (95% *CI*: 0.72%, 1.91%), 231.62 (43.45, 419.78) mL/s, and 131.64 (37.54, 225.74) mL/s respectively, when all chemicals were at 75th percentile. In the quantile-based g-computation, each quartile increase in mixture was associated with 104.35 (95% *CI*: 40.67, 168.02) mL, 1.16% (2.11%, 22.40%), 294.90 (78.37, 511.43) mL/s, 168.44 (41.66, 295.22) mL/s decrease in the FEV_1_, FEV_1_/forced vital capacity, peak expiratory flow, and forced expiratory flow between 25% and 75%, respectively. 2-Hydroxyphenanthrene, 3-Hydroxyfluorene, and cadmium were leading contributors to the above associations. WBC mediated 8.22%-23.90% of association between PAHs and lung function.

**Conclusions:**

Co-exposure of PAHs and metals impairs lung function, and WBC could partially mediate this relationship. Our findings elucidate co-exposure effects of environmental mixtures on respiratory health and underlying mechanisms, suggesting that focusing on highly prioritized toxicants would effectively attenuate adverse effects.

**Supplementary Information:**

The online version contains supplementary material available at 10.1186/s12890-024-03173-9.

## Introduction

Lung function reduction is an early indicator of numerous respiratory diseases [1], and it is also associated with a higher incidence and mortality of cardiovascular disease [2, 3]. Mounting evidence has generally supported that genetic variants [4], diet [5], infection [6], and environmental pollution [7] could influence lung function. Environmental pollution has become a pressing concern due to its complex and ubiquitous exposure. Environmental factors, rather than genetic factors, may be the main contributors to the impairment of lung function among adults, as adults are in the decline phase of their lung function trajectory. Therefore, recent studies have focused on identifying environmental risk factors for lung function reduction.

Both polycyclic aromatic hydrocarbons (PAHs) and metals coexist in atmospheric pollutants. PAHs come from two sources: natural sources and man-made sources. Man-made sources are the main ones, originating from daily life (traffic-related emissions, smoking, cooking and heating) and industrial activities (coal-fired power plants, electronic waste recycling, asphalt, foundries and blast furnaces) [8]. Metals mainly come from metal mining; combustion of petroleum, diesel and coal; geochemical processes [9]. In the general population, main routes of PAHs and metals exposure include inhaling ambient air pollution, consuming contaminated food, and encountering dermal contact [10, 11]. PAHs do not appear as a single compound, but rather as a mixture of various PAHs [12]. Among these, 16 derivatives of PAHs are considered as priority pollutant PAHs by the United States Environmental Protection Agency. These 16 derivatives are just a small part of PAHs, and their volatility generally decreases with the increase in the number of benzene ring. These PAHs include acenaphthene, acenaphthene, anthracene, benzo(a)anthracene, benzo(a)pyrene, benzo(b)fluoranthene, benzo(ghi)perylene, benzo(k)fluoranthene, dibenzo(a,h)anthracene 3-cd fluoranthene [13]. After human which enables to PAHs, PAHs can diffuse through the cell membrane due to their lipophilicity. There are three known metabolic pathways of PAHs: CYP1A1/1B1 and epoxide hydrolase pathway, CYP peroxidase pathway, and aldosterone reductase pathway. Currently, PAHs and metals pollution have become serious environmental problems [14, 15], and epidemiological evidence has generally supported that exposure to PAHs and metals was related to adverse respiratory outcomes. Among 1,000 individuals aged 22–25 years old from Sweden, researchers found that urinary metabolites of phenanthrene and fluorene were negatively associated with forced expiratory volume in 1 s (FEV_1_), forced vital capacity (FVC), as well as 1-hydroxypyrene (1-OHP) with FEV_1_/FVC [16]. Then, a birth cohort study conducted in 294 non-asthmatic children found that exposure to higher levels of PAHs was related to lower FVC and FEV_1_ levels [17]. Numerous studies have demonstrated that elevated concentrations of metals could impair lung function. In a recent study conducted in 382 adults from Gansu province, China, heavy metal exposure, such as antimony, mercury, and manganese, was associated with reductions in FEV_1_% and FVC%; but there was a positive association with molybdenum [18]. Among 186 welders from Anhui Province, China, researchers found a significant association between multi-metal mixtures and lung function reduction, and key contributors included lead (Pb), cadmium (Cd), nickel, and strontium [19].

Although numerous studies have examined the impact of PAHs or metals on respiratory health, research of their combined effects remains limited. In the real world, human beings are simultaneously exposed to multiple environmental pollutants. To estimate co-exposure effects of these pollutants, several innovative statistical methods have been developed, including shrinkage and selection operator (LASSO), Bayesian kernel machine regression (BKMR), and quantile-based g-computation (qgcomp).

Circulating white blood cell (WBC) count is a marker of systemic inflammation, and it was related to lung function reduction and airflow obstruction in both occupational and general population [20, 21]. Experimental studies in rats also supported that exposure to diesel engine exhaust, which was coated with PAHs, resulted in allergic lung inflammation, with an increase in WBC being one of the effective indicators [22]. Inhalable dust particles contain various elements (including PAHs and metals) and affect immune cells, which may lead to lung function reduction [21]. Based on a review of previous studies, circulating WBC may play a mediating role in the relationship between co-exposure to PAHs and metals and lung function reduction [20–24].

The objectives of this study were to explore combined exposure effects of PAHs and metals on lung function, and to investigate mediating role of WBC in the aforementioned relationship among 1,123 adults from the general United States population. Four commonly used statistical methods were applied to analyze co-exposure effect of PAHs and metals, which would improve the robustness and convincement of our findings.

## Methods and methods

### Study population

NHANES 2011–2012 is a national cross-sectional survey aimed to assess the health and nutritional status of the American, investigate the prevalence of common diseases, formulate national standards, and conduct health education. It adopts multi-stage sampling to select representative samples throughout the country: all counties in the United States are divided into 15 groups based on their basic characteristics, and one county is selected from each group, resulting in a total of 15 counties being chosen. Then, 20 to 24 blocks are selected in each county and approximately 30 families are selected in each block. In each family, family members are extracted by a computer algorithm. In 2011–2012, 9,756 subjects were included in NHANES, including 5,560 subjects aged over 20 years old (children do not have smoking data). In order to understand the health and nutritional status of Asian Americans, NHANES over-sampled Asian Americans in 2011–2012. The personal information and health status of the respondents were primarily obtained through family interviews and physical examinations. Information on detailed methods, protocols and definitions could be found in the NHANES website (https://www.cdc.gov/nchs/nhanes/index.htm). In the present study, 8,633 participants were excluded due to missing data on height (*n* = 1,141), smoking (*n* = 3,371), drinking (*n* = 610), family poverty-income ratio (PIR) (*n* = 365), urinary PAHs or metals information (*n* = 2,905), spirometry (*n* = 211), or participants with lung function quality attribute of D-F (*n* = 30). Finally, 1,123 participants aged 20–80 years were included (Fig. S1). Written informed consent was obtained from all participants, and the protocols for NHANES 2011–2012 were approved by the institutional review board of the National Center for Health Statistics (no. 2011–17).

### Spirometry assessment

The Ohio 822/827 dry-roll volume spirometer was used by technicians to assess lung function. Participants maintained a standing position, wore a nose clip during the test, inhaled as hard as possible to fill their lungs with air, and then exhaled as quickly as possible. Participants aged 11–79 were required to exhale for at least 6 s. Based on the guidelines provided by the American Thoracic Society (ATS)/European Respiratory Society [25], lung functions were classified into A-F. A-B levels indicate that data quality meets the standard of ATS and has good repeatability, grade C indicates that a portion of data could fulfill ATS standard, while grades D-F show that the data should be cautiously used or are invalid. According to the recommendations provided in the literature, statistical analysis was conducted among participants with lung function data rated A to C measured without inhalation of bronchodilators [26]. Three maneuvers were performed by the subjects and the best one selected. In our study, five lung function indices, including FEV_1_ (mL), FVC (mL), FEV_1_/FVC, forced expiratory flow between 25% and 75% (FEF_25-75%_) (mL/s), and peak expiratory flow (PEF) (mL/s), were used in the analyses.

### Determination of urinary PAHs

Urinary levels of hydroxyl-PAHs (OH-PAHs) were analyzed using an Agilent 7000A tandem mass spectrometer coupled with an Agilent 7890A gas chromatograph (Agilent Technologies, Santa Clara, CA). Enzymatic hydrolysis was utilized to break up glucuronidated/sulfated OH-PAH metabolites, followed by extraction, and derivatization [27, 28]. In NHANES 2011–2012, the analyzed components were consisted of 10 OH-PAHs, included two derivatives of hydroxynaphthalene (1- and 2-OHNa), three derivatives of hydroxyfluorene (2-, 3-, and 9-OHFlu), four derivatives of hydroxyphenanthrene (1-, 2-, 3-, and 4-OHPh), and 1-OHP. Table S1 showed that limits of detection (LOD) of 10 OH-PAHs ranged from 10 to 44 ng/L. In case of concentration was < LOD, it was replaced with LOD/sqrt (2). Detection rates of most OH-PAHs were > 95% except 4-OHPh (76.48%, Table S1). Urinary creatinine was used to account for urine dilution, and urinary concentrations of OH-PAHs were expressed as ng/mmol creatinine.

### Measurement of urinary metals

Urinary concentrations of 11 metals (barium, Ba; cadmium, Cd; cobalt, Co; cesium, Cs; molybdenum, Mo; manganese, Mn; lead, Pb; antimony, Sb; thallium, Tl; tungsten, W; uranium, U) were quantified utilizing inductively coupled plasma dynamic reaction cell mass spectrometry (ICP-DRC-MS). The urine sample was introduced into the ICP system through a nebulizer and spray chamber. The radio-frequency power applied to the flowing argon generated a high-temperature plasma region (6,000–8,000 ºK). In this region, the sample is atomized and subsequently ionized. The ions, along with the argon carrier gas, enter the mass spectrometer via an interface that separates the ICP from the mass spectrometer. The mass spectrometer enables the detection of ions of each mass in a rapid sequence, thereby facilitating the determination of a single isotope of element. The electrical signal generated by ion detection is converted into digital information, which indicates the ion intensity and elemental concentration [29, 30]. LOD for 11 metals ranged from 3.3 to 990 ng/L and detection rate ranged from 69.13% to 100% (Table S1). In sample with concentration < LOD, it was replaced with LOD/sqrt (2). Correction for urinary dilution was achieved by using urinary creatinine, and metal concentrations were subsequently expressed as ng/mmol creatinine.

### Covariates

A questionnaire was used to obtain sociodemographic data, including gender, age, race (Mexican–American, Other Hispanic, non-Hispanic white, non-Hispanic black, and other race), smoking and drinking status. Based on their smoking habits, participants were divided into two categories: smokers, defined as individuals who have smoked ≥ 100 cigarettes during their lifetime; otherwise, they were classified as non-smokers. Drinkers was defined based on self-reported positive answer to the question: “Had at least 12 alcohol drinks/1 year?” Standing height was assessed using a stadiometer. Socioeconomic status was assessed using the PIR, which was determined by dividing the annual family income by poverty standard of family population in the state of participants, based on federal standards. In this study, PIR was recoded as a dichotomous variable: PIR < 5 (low income) or ≥ 5 (high income). Details on the questionnaires are described in the Supplementary Material.

### Statistical analysis

Descriptive statistics were carried out using frequency and proportion for categorical variables, and mean and standard error (SE) or median with interquartile range for continuous variables. Because of the skewed distribution of PAHs and metals, they were log_10_-transformed. Pearson correlation coefficients between OH-PAHs and metals were visualized via a heat map.

Firstly, we conducted a single-exposure model using generalized linear model (GLM), and 21 chemicals were separately included in the model as continuous independent variables to calculate coefficient and 95% confidence intervals (CI) for five lung function indices, with adjustment for age, gender, race, height, family PIR, smoking and drinking status. Moreover, stratified analyses by gender were performed.

Then, LASSO penalized regression analysis was used to select significant chemicals associated with lung function by constructing penalty function. This approach reduces the complexity of the model and avoids the overfitting problem caused by the correlation between exposures [31]. With tenfold cross-validation, the best λ value with the smallest cross-validation error, i.e. minimum mean squared error, was chosen [32].

BKMR utilizes a Gaussian kernel function to flexibly evaluate exposure–response function, which enables the identification of non-linear and non-additive associations, as well as interactions between multiple metabolites [33]. Using a probabilistic link function for associations between mixed exposures and continuous outcomes [34], BKMR was implemented to quantify the association between 21 chemicals and lung function. This method allows for the selection of variables either component-wise or hierarchical manner. In order to evaluate the importance of each pollutant, posterior inclusion probabilities (PIP) are assessed, and pollutants with PIP > 0.500 are regarded as significant. Here, we evaluated combined and individual effects using 100,000 iterations of a Markov Chain Monte Carlo sampler. By fixing the 21 chemicals at a specific quantile, we plotted the cumulative effect, single exposure effect, and univariate dose–response relationship of chemicals with lung function [35]. Finally, a summary of single chemical interactions was visualized.

Qgcomp classified all chemicals into quartiles and used these quantified exposures as continuous variables to fit the linear regression model [36]. The mixing effect on lung function was studied by considering the simultaneous increase of all chemicals by a quartile. A positive or negative weight was assigned to each chemical, representing its contribution to the overall effect.

Mediation analysis aimed to explore the mediating role of WBC in the associations between pollutants and lung function. The model utilized 5,000 bootstraps to ensure robustness. Direct effects of PAHs and metals on lung function were examined using a GLM model. Indirect effects were calculated by assessing the relationships of PAHs and metals with WBC, and of WBC with lung function, also using a GLM model. The proportion mediated by WBC was determined by comparing the indirect effect to the overall effect, which encompassed both direct and indirect effects.

We performed sensitivity analyses by excluding participants with asthma and chronic obstructive pulmonary disease (COPD), defined as post-bronchodilator FEV_1_/FVC < 0.70.

GLM analyses were conducted with SAS (version 9.4; SAS Institute Inc., NC, USA). LASSO, BKMR, qgcomp, and mediation regression were conducted using the “glmnet”, “bkmr”, “qgcomp”, and “mediation” packages, respectively (R software version 4.0.3). A two-sided *P* < 0.05 was regarded as statistically significant.

## Results

### Characteristics of participants

General characteristics of study population were shown in Table 1, and characteristics of the whole adult population and the subsample investigated in this study (*n* = 1,123) were comparable (Table S2). Study population (*n* = 1,123) was comprised of 528 (49.50%) females and 595 (50.50%) males. Mean ± SE for age was 44.93 ± 1.02 years old, and for height was 169.33 ± 0.38 cm. There was 69.50% non-Hispanic White, followed by 10.60% non-Hispanic Black, 7.40% Others, 6.30% Mexican American, and 6.20% Other Hispanic. Mean ± SE for FVC, FEV_1_, FEV_1_/FVC, PEF, and FEF_25-75%_ were 4,132.41 ± 42.48 mL, 3,221.70 ± 37.84 mL, 78.05% ± 0.44, 8,445.63 ± 118.37 mL/s, and 3,005.46 ± 62.77 mL/s, respectively.

**Table 1:** Baseline characteristics, urinary levels of PAHs and metals metabolites, and lung function level among the study participants (*n* = 1,123)

Variables	Values
**General characteristics**	
Age (years)	44.93 ± 1.02
Gender	
Male	595 (50.50)
Female	528 (49.50)
Height (cm)	169.33 ± 0.38
Race	
Mexican American	98 (6.30)
Other Hispanic	115 (6.20)
Non-Hispanic White	422 (69.50)
Non-Hispanic Black	295 (10.60)
Others	193 (7.40)
Cigarette smoking	
Never	646 (56.60)
Ever	477 (43.40)
Alcohol drinking	
Never	861 (83.8)
Ever	262 (16.20)
Family PIR	
0–4.99	912 (76.90)
≥ 5	211 (23.10)
**Urinary PAH (*ng*/mmol creatinine)**	
2-OHFlu	24.03 (9.43, 259.87)
3-OHFlu	8.92 (2.74, 136.26)
9-OHFlu	30.97 (9.11, 170.31)
1-OHNa	185.76 (38.67, 2,411.46)
2-OHNa	524.37 (134.00, 2,660.82)
1-OHPh	15.74 (5.86, 61.40)
2-OHPh	7.71 (2.89, 33.18)
3-OHPh	7.38 (2.50, 39.96)
4-OHPh	2.51 (0.75, 13.26)
1-OHP	12.76 (3.82, 74.76)
**Urinary metals (*ng*/mmol creatinine)**	
Ba	152.76 (33.69, 634.34)
Cd	22.15 (5.12, 99.79)
Co	35.39 (15.00, 124.76)
Cs	487.02 (213.68, 1,155.51)
Mo	4,264.60 (1,554.21, 11,781.25)
Mn	15.13 (3.98, 61.69)
Pb	45.35 (14.14, 164.86)
Sb	6.33 (2.26, 24.36)
Tl	18.92 (8.29, 49.59)
W	7.79 (2.54, 37.54)
U	0.69 (0.22, 3.22)
**Lung function indices**	
FVC (mL)	4,132.66 ± 42.48
FEV_1_ (mL)	3,221.70 ± 37.84
FEV_1_/FVC (100%)	78.05 ± 0.44
PEF (mL/s)	8,445.63 ± 118.37
FEF_25-75%_ (mL/s)	3,005.46 ± 62.77
**WBC (1000 cells/μL)**	6.98 ± 0.11

Continuous variables were presented as mean ± SE or median (5th, 95th percentiles). Categorical variables were presented as *n* (%)

*Abbreviations: 2-OHFlu* 2-Hydroxyfluorene, *3-OHFlu* 3-Hydroxyfluorene, *1-OHNa* 1-Hydroxynapthalene, *2-OHNa* 2-Hydroxynapthalene, *9-OHFlu* 9-Hydroxyfluorene, *1-OHPh* 1-Hydroxyphenanthrene, *2-OHPh* 2-Hydroxyphenanthrene, *3-OHPh* 3-Hydroxyphenanthrene, *4-OHPh* 4-Hydroxyphenanthrene, *1-OHP* 1-Hydroxypyrene, *Ba* barium, *Cd* cadmium, *Co* cobalt, *Cs* cesium, *Mo* molybdenum, *Mn* manganese, *Pb* lead, *PIR* poverty-income ratio, *Sb* antimony, *Tl* thallium, *W* tungsten, *U* uranium, *FVC* forced vital capacity, *FEV*_*1*_ forced expiratory volume in 1 s, *PEF* peak expiratory flow, *FEF*_*25-75%*_ forced expiratory flow between 25% and 75%

### Distribution and correlation of urinary PAHs and metals

Median concentrations of OH-PAHs and metals ranged from 2.51 to 522.70 ng/mmol creatinine and 0.69–4,264.60 ng/mmol creatinine, respectively (Table 1). Fig. S2 depicted the Pearson correlation coefficients among the 21 log_10_-transformed chemicals. Strong positive correlations (> 0.70) were found in 19 pairs of PAHs, like 3-OHFlu and 2-OHFlu (*r* = 0.95), 3-OHPh and 2-OHPh (*r* = 0.90), and 1-OHPh and 2-OHPh (*r* = 0.85).

### Associations of PAHs, metals, and circulating WBC with lung function by GLM

The 1-OHNa and 2-OHNa were negatively associated with FEV_1_/FVC and PEF (obstructive pattern) and FEF_25-75%_ (small airway dysfunction), and 3-OHFlu, 2-OHFlu and 3-OHPh were negatively associated with FEV_1_, FEV_1_/FVC and PEF (obstructive pattern), as well as FEF_25-75%_ (small airway dysfunction). There were also significant associations of 2-OHPh and 1-OHP with decreased FEV_1_ and FEV_1_/FVC (obstructive pattern). Cd was negatively associated with FEV_1_, FEV_1_/FVC and PEF (obstructive pattern), as well as FEF_25-75%_ (small airway dysfunction). However, Mo was negatively associated with FVC but positively associated with FEV_1_/FVC (restrictive pattern). Besides, increased WBC count was related to decreased FVC, FEV_1_, PEF, and FEF_25-75%_ (Table 2).

**Table 2:** Associations of urinary PAHs metabolites, urinary metals, and circulating white blood cell counts with lung function in NHANES 2011–2012

Exposures	FVC, mL		FEV_1_, mL		FEV_1_/FVC (%)		PEF, mL/s		FEF_25–75%_, mL/s	
	*β* (95% *CI*)	*P*	*β* (95% *CI*)	*P*	*β* (95% *CI*)	*P*	*β* (95% *CI*)	*P*	*β* (95% *CI*)	*P*
**Urinary PAHs**										
2-OHFlu	-28.01 (-149.65, 93.63)	0.633	-158.04 (-256.86, -59.23)	**0.004**	-3.4 ( -5.1, -1.6)	** < 0.001**	-522.95 (-881.59, -164.32)	**0.007**	-354.91 (-528.10, -181.72)	** < 0.001**
3-OHFlu	-4.92 (-103.92, 94.07)	0.918	-118.23 (-190.75, -45.72)	**0.003**	-2.9 (-4.3, -1.5)	** < 0.001**	-415.07 (-708.46, -121.67)	**0.008**	-285.07 (-398.44, -171.69)	** < 0.001**
9-OHFlu	-121.98 (-277.05, 33.10)	0.115	-159.24 (-297.72, -20.76)	**0.027**	-1.6 (-3.5, 0.39)	0.110	-342.10 (-782.61, 98.41)	0.120	-190.60 (-465.92, 84.71)	0.171
1-OHNa	33.70 (-63.18, 130.58)	0.473	-46.73 (-129.33,35.88)	0.249	-1.9 (-2.6, -1.1)	** < 0.001**	-291.76 (-508.23, -77.27)	**0.010**	-178.01 (-299.04, -56.97)	**0.007**
2-OHNa	37.75 (-48.58, 124.08)	0.369	-100.72 (-207.07, 5.62)	0.062	-3.1 (-5.2, -1.0)	**0.007**	-429.90 (-826.01, -33.79)	**0.035**	-273.81 (-509.00, -38.62)	**0.025**
1-OHPh	-72.98 (-211.58, 65.63)	0.282	-126.75 (-242.17, -11.32)	**0.033**	-1.2 (-3.3, 0.8)	0.212	-138.69 (-511.46, 234.09)	0.367	-160.77 (-373.85, 52.31)	0.130
2-OHPh	-116.42 (-259.22, 26.38)	0.104	-186.81 (-325.03, -48.59)	**0.011**	-2.0 (-4.0, -0.004)	**0.050**	-378.09 (-820.93, 64.76)	0.089	-298.13 (-515.14, -81.11)	**0.010**
3-OHPh	-39.34 (-161.98, 83.29)	0.508	-134.60 (-239.77, -29.43)	**0.015**	-2.3 (-4.2, -0.5)	**0.017**	-367.83 (-720.18, -15.48)	**0.042**	-269.51 (-460.62, -78.41)	**0.009**
4-OHPh	-95.23 (-217.62, 27.16)	0.119	-160.23 (-279.02, -41.43)	**0.011**	-1.9 (-4.2, 0.4)	0.105	-128.80 (-533.01, 275.40)	0.510	-231.75 (-459.64, -3.86)	**0.047**
1-OHP	-58.81 (-188.46, 70.85)	0.352	-139.69 (-248.59, -30.78)	**0.015**	-2.1 (-3.9, -0.25)	**0.028**	-313.47 (-654.46, 27.51)	0.057	-245.33 (-425.74, -64.91)	**0.011**
∑OHFlu	-75.20 (-218.85, 66.45)	0.278	-178.16 (-295.01, -61.30)	**0.005**	-2.4 (-3.5, -1.2)	** < 0.001**	-484.59 (-877.79, -91.39)	**0.019**	-315.61 (-541.59, -88.84)	**0.009**
∑OHNa	37.12 (-76.72, 150.95)	0.466	-89.34 (-196.21, 17.54)	0.098	-2.2 (-3.1, -1.4)	** < 0.001**	-432.77 (-729.75, -135.78)	**0.007**	-273.03 (-459.56, -86.49)	**0.007**
∑OHPh	-82.82 (-225.27, 59.64)	0.711	-160.94 (-288.75, -33.13)	**0.017**	-1.8 (-3.2, -0.4)	**0.014**	-274.33 (-680.94, 131.79)	0.172	-249.71 (-474.54, -24.87)	**0.032**
∑PAH	18.52 (-104.85, 141.89)	0.755	-107.72 (-221.12, 5.68)	0.061	-2.9 (-4.4, -1.4)	** < 0.001**	-462.71 (-782.21, -143.20)	**0.007**	-286.76 (-486.53, -86.99)	**0.008**
**Urinary metals**										
Ba	-99.76 (-266.51, 66.99)	0.224	-71.52 (-209.91, 66.87)	0.291	0.5 (-1.4, 2.4)	0.581	-132.74 (-521.77, 256.29)	0.499	2.87(-191.25, 197.00)	0.976
Cd	-30.01 (-147.31, 87.29)	0.596	-182.67 (-285.82, -79.51)	**0.002**	-4.1 (-5.9, -2.3)	** < 0.001**	-381.33 (-728.07, -34.58)	**0.033**	-397.57(-614.52, -180.61)	**0.001**
Co	-184.78 (-385.99, 16.43)	0.070	-152.45 (-322.55, 17.64)	0.076	0.02 (-2.8, 2.8)	0.987	-220.53 (-841.31, 400.26)	0.464	-94.46 (-462.73, 273.82)	0.595
Cs	2.19 (-209.44, 213.83)	0.983	15.17 (-151.74, 182.08)	0.850	0.3 (-1.4, 2.1)	0.691	291.57 (-167.93, 751.08)	0.198	48.94 (-240.71, 338.60)	0.726
Mo	-221.91 (-381.47, -62.35)	**0.009**	-60.99 (-221.78, 99.811)	0.435	2.8 (0.4, 5.2)	**0.025**	48.66 (-488.14, 585.46)	0.851	169.62 (-183.88, 523.13)	0.326
Mn	-62.02 (-181.42, 57.38)	0.288	-17.89 (-123.55, 87.77)	0.725	0.39 (-1.4, 2.2)	0.661	257.82 (-201.15, 716.79)	0.252	38.18 (-156.95, 233.31)	0.685
Pb	60.61 (-145.96, 267.17)	0.544	-20.08 (-218.92, 178.77)	0.834	1.80 (3.2, -0.4)	**0.016**	-254.09 (-733.80, 225.63)	0.279	-121.97 (-445.80, 201.86)	0.438
Sb	-56.27 (-201.99, 89.45)	0.427	-23.51 (-132.04, 85.01)	0.653	0.08 (-1.8, 1.9)	0.932	131.09 (-252.54, 514.72)	0.481	73.29 (-160.75, 307.33)	0.518
Tl	67.65 (-164.62, 299.92)	0.547	98.07 (-76.38, 272.51)	0.252	1.6 (1.2, 4.4)	0.251	807.12 (182.78, 1431.46)	**0.014**	255.10 (-99.63, 609.83)	0.148
W	-109.47 (-239.04, 20.09)	0.093	-50.34 (-188.66, 87.97)	0.453	0.46 (-1.2, 2.1)	0.554	-183.69 (-507.74, 140.36)	0.248	22.64 (-256.80, 302.07)	0.866
U	-119.54 (-286.96, 47.87)	0.15	-141.53 (-239.19, -43.87)	**0.007**	-1.3 (-3.5, 0.90)	0.232	-349.12 (-738.52, 40.28)	0.076	-111.40 (-300.75, 77.94)	0.231
**WBC**	-31.47 (-56.56, -6.38)	**0.017**	-39.52 (-66.93, -12.10)	**0.007**	-0.3 (-0.7, 0.06)	0.093	-114.58 (-195.48, -33.69)	**0.008**	-52.99 (-101.41, -4.57)	**0.034**

Log_10_-transformed, creatinine-corrected urinary concentration of each PAHs metabolite and metal and circulating white blood cell counts were modeled as continuous variables to calculate coefficient and 95% confidence intervals (*CI*) for lung function. Generalized linear model was for gender, age, race, height, PIR, smoking and drinking status. ∑OHFlu, sum of 2-OHFlu, 3-OHFlu, and 9-OHFlu. ∑OHNa, sum of 1-OHNa and 2-OHNa. ∑OHPh, sum of 1-OHPh, 2-OHPh, 3-OHPh, and 4-OHPh. ∑PAH, sum of all the 10 PAHs metabolites

### Stratified GLM analysis by gender

Co-gender interaction effects on FVC and FEV_1_ were observed (*P* for interaction was 0.033 and 0.027, respectively), and there was a negative association in male but not female. Besides, interactions of 1-OHPh, Ba, and U with gender on PEF were found (*P* for interaction was 0.040, 0.008, and 0.044, respectively), and male had larger decrease in PEF than female when exposed to 1-OHPh, Ba, and U (Table S3-S7).

### LASSO penalized regression analyses for multi-pollutant

**Fig. 1:**
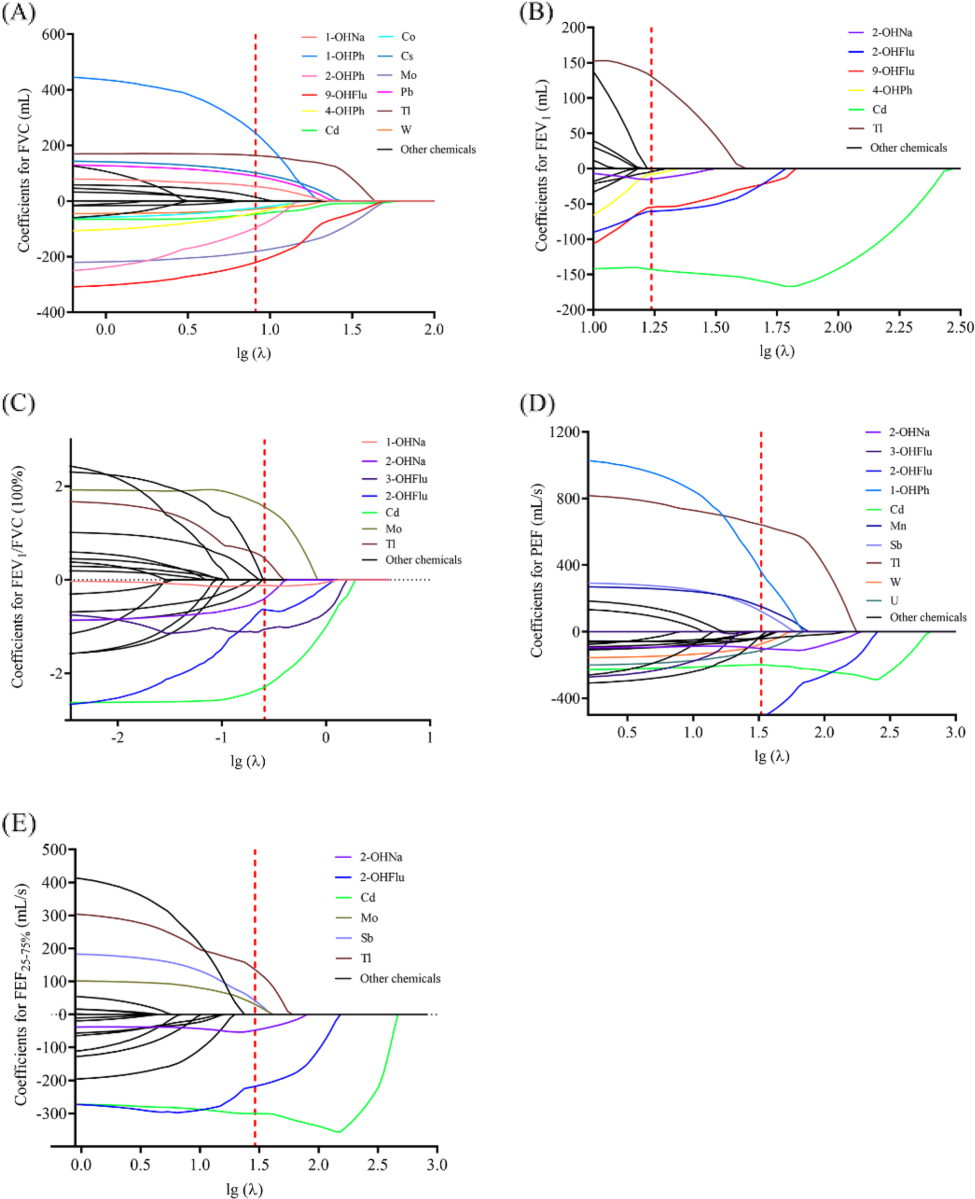
Association between PAHs and metals and lung function indices by LASSO regression models. Notes: In the LASSO regression model, log_10_-transformed PAHs and metals were included as independent variables, lung function indices were included as the dependent variables, with adjustment for age, gender, race, height, family poverty-income ratio, drinking status and smoking status. The red dotted line represented the optimal value of λ with minimum mean squared error. Abbreviations: 2-OHFlu, 2-Hydroxyfluorene; 3-OHFlu, 3-Hydroxyfluorene; 9-OHFlu, 9-Hydroxyfluorene; 1-OHNa, 1-Hydroxynapthalene; 2-OHNa, 2-Hydroxynapthalene; 1-OHPh, 1-Hydroxyphenanthrene; 4-OHPh, 4-Hydroxyphenanthrene; Cd, cadmium; Cs, cobalt; FEF_25-75%_, forced expiratory flow between 25% and 75%; FEV_1_, forced expiratory volume in 1 s; FVC, forced vital capacity; Mo, molybdenum; Mn, manganese; Pb, lead; PEF, peak expiratory flow; Sb, antimony; Tl, thallium; W, tungsten; U, uranium

Considering dimensionality and collinearity of chemicals, LASSO regression was carried out. The 8.17, 17.28, 0.257, 33.02, and 29.14 were the best λ for FVC, FEV_1_, FEV_1_/FVC, PEF, and FEF_25-75%_, respectively (Fig. 1).

LASSO model produced non-zero coefficients for 12 chemicals (1-OHNa, 1-OHPh, 2-OHPh, 9-OHFlu, 4-OHPh, Cd, Co, Cs, Mo, Pb, Tl, W) with FVC, 6 chemicals (2-OHNa, 2-OHFlu, 9-OHFlu, 4-OHPh, Cd, and Tl) with FEV_1_, 6 chemicals (1-OHNa, 2-OHNa, 3-OHFlu, 2-OHFlu, Cd, and Mo) with FEV_1_/FVC, 10 chemicals (2-OHNa, 3-OHFlu, 2-OHFlu, 1-OHPh, Cd, Mn, Sb, Tl, W, and U) with PEF, and 6 chemicals (2-OHNa, 2-OHFlu, Cd, Mo, Sb, and Tl) with FEF_25-75%_ after adjusting for all the covariates.

### BKMR analyses for multi-pollutants

**Fig. 2:**
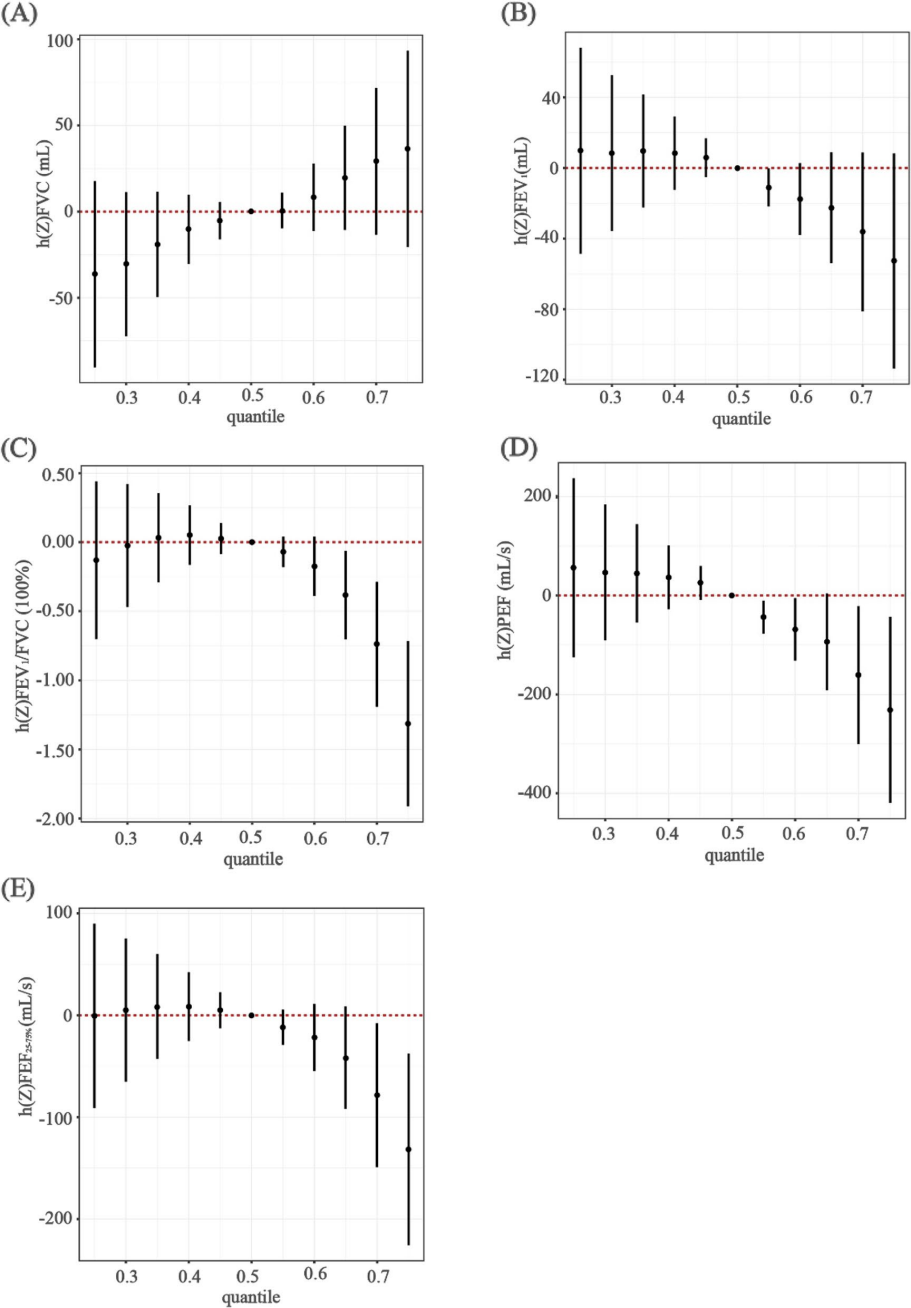
Associations between the overall element mixture and lung function indices by Bayesian kernel machine regression analyses. Note: In the Bayesian kernel machine regression analyses, age, gender, race, height, family poverty-income ratio, drinking status and smoking status were adjusted. Abbreviations: FEF_25-75%_, forced expiratory flow between 25% and 75%; FEV_1_, forced expiratory volume in 1 s; FVC, forced vital capacity; PEF, peak expiratory flow

The results of BKMR model for FVC revealed the PIP of Cs (0.948) was the highest, followed by 1-OHPh (0.854) and 2-OHPh (0.835). For FEV_1_, 1-OHPh, Cs, 2-OHFlu, 2-OHPh, Cd, Tl, and 9-OHFlu were significant contributors (all PIPs > 0.500). Cd (PIP = 0.987) and 3-OHFlu (PIP = 0.947) were selected for FEV_1_/FVC. 2-OHFlu (PIP = 0.963), 1-OHPh (PIP = 0.903), and Tl (PIP = 0.585) were significant contributors to PEF. For FEF_25-75%_, BKMR model revealed that the PIP of 3-OHFlu (0.813) was the highest, followed by Cd (0.590) (Table S8). We then estimated overall effect on lung function. FEV_1_/FVC, PEF, and FEF_25-75%_ showed a downward trend with the cumulative level of chemical increase (Fig. 2). Comparing to all chemicals fixed at their median value, FEV_1_/FVC, PEF and FEF_25-75%_ decreased by 1.31% (95% *CI*: 0.72%, 1.91%), 231.62 (95% *CI*: 43.45, 419.78) mL/s and 131.64 (95% *CI*: 37.54, 225.74) mL/s respectively, when all chemicals were at 75th percentile (obstructive pattern and small airway dysfunction). Significant exposure–response relationships were observed between 2-OHFlu and FEV_1_ and PEF (obstructive pattern). There were also significant relationships of 2-OHPh with FEV_1_ and of 1-OHPh with PEF (obstructive pattern), as well as 3-OHFlu and Cd with FEF_25-75%_ (small airway dysfunction). We observed a non-linear relationship between 3-OHFlu and Cd and FEV_1_/FVC (obstructive pattern, Fig. S3). In Fig. S4, we examined independent effect of single exposure when other chemicals were fixed at 25th, 50th, or 75th. We observed that Cd was significantly associated with decreased FEV_1_, FEV_1_/FVC, and FEF_25-75%_ (obstructive pattern and small airway dysfunction). Additionally, we observed significant effects of 3-OHFlu on FEV_1_/FVC, and 2-OHFlu on PEF (obstructive pattern). No significant single chemical interaction was observed (Fig. S5).

### Associations of PAHs and metals with lung function in the qgcomp for multi-pollutant

**Fig. 3:**
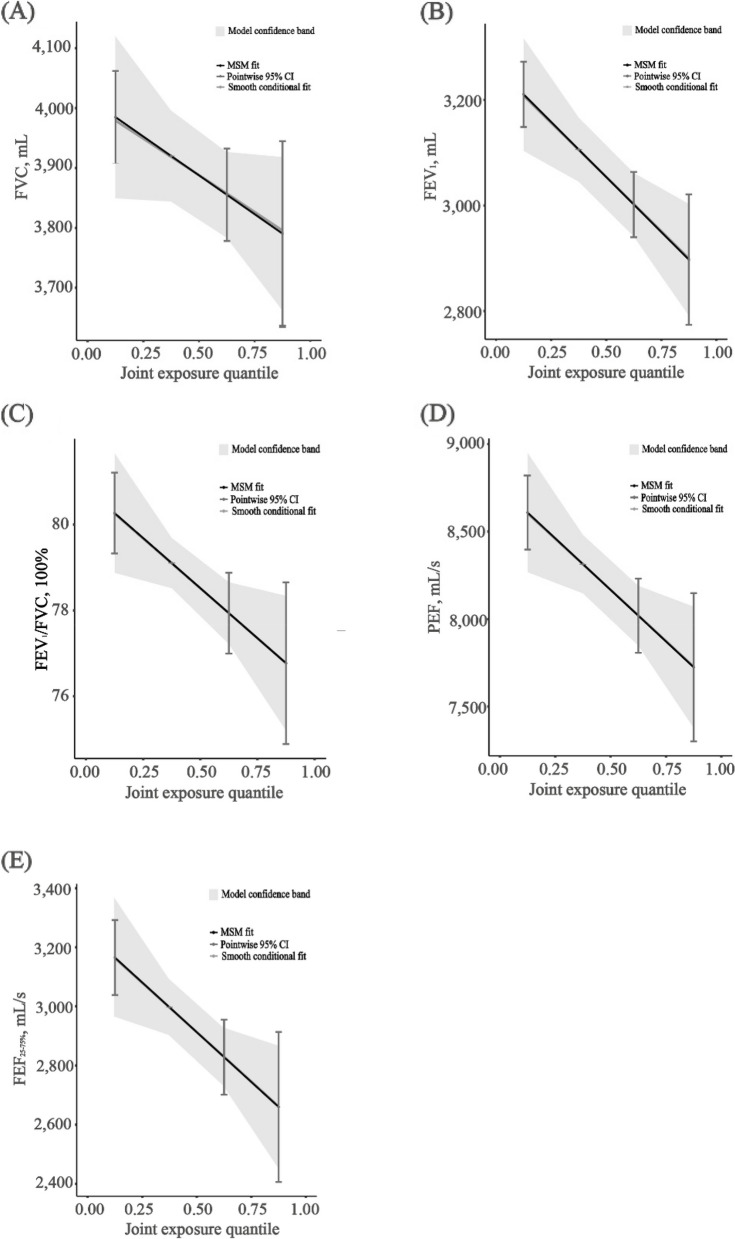
Mixture effects of PAHs and metals on FEV_1_, FEV_1_/FVC, FEF_25–75%_, and PEF levels by quantile-based g-computation analyses. Note: Covariates include age, gender, race, height, family poverty-income ratio, drinking status and smoking status. Abbreviations: FEF_25-75%_, forced expiratory flow between 25% and 75%; FEV_1_, forced expiratory volume in 1 s; FVC, forced vital capacity; PEF, peak expiratory flow

Qgcomp showed that exposure to a mixture of PAHs and metals was inversely associated with FEV_1_, FEV_1_/FVC, PEF and FEF_25-75%_ (obstructive pattern and small airway dysfunction, Fig. 3). Each quartile increased in chemicals mixture was associated with 104.35 (95% *CI*: 40.67, 168.02) mL, 1.16% (95% *CI*: 22.40%, 2.11%), 294.90 (95% *CI*: 78.37, 511.43) mL/s, and 168.44 (95% *CI*: 41.66, 295.22) mL/s decrease in the FEV_1_, FEV_1_/FVC, PEF, and FEF_25-75%_, respectively. In the qgcomp, most exposures were given negative weights related to lung function, which aligned with the overall associations (Fig. S6). 2-OHPh, Mo, and Co were the major contributors for the negative association with FVC, with the weights of 39.94%, 17.53%, and 13.17%, respectively. 2-OHPh, Cd, and Co accounted for 43.37%, 12.12%, and 11.71 of the negative effect on FEV_1_, respectively; and Cd, 3-OHFlu, and Co accounted for 20.75%, 15.64%, and 13.31% of the negative effect on FEV_1_/FVC, respectively. 2-OHPh, Co and Cd accounted for 26.41%, 14.42%, and 11.86% of the negative effect on PEF, respectively; and Cd, 2-OHPh and Co contributed 22.29%, 21.87%, and 13.47% to the negative effect on FEF_25-75%_, respectively_._

### Mediation analysis

As show in table S9, 2-OHFlu, 3-OHFlu, 2-OHNa, ∑OHFlu (sum of 3-OHFlu, 2-OHFlu and 9-OHFlu), ∑OHNa (sum of 1-OHNa and 2-OHNa), ∑PAH (sum of all the 10 PAHs metabolites) and Co were significantly associated with WBC. Besides, WBC was also associated with FEV_1_, PEF and FEF_25-75%_. Therefore, we explored the mediating role of WBC in the relationship of 2-OHFlu, 3-OHFlu, 2-OHNa, ∑OHFlu, ∑OHNa, ∑PAH and Co with FEV_1_, PEF and FEF_25-75%_. The associations of ∑OHFlu with FEV_1_ was significantly mediated by WBC (mediated proportion was 16.30%). And WBC also played a mediating role in the relationships of 2-OHFlu and 3-OHFlu with FEV_1_, mediated proportion was 20.67% and 17.80%, respectively. We found that among total effects of 2-OHFlu, 3-OHFlu, 2-OHNa, ∑OHFlu, ∑OHNa, and ∑PAH on PEF, 15.66%, 12.77%, 23.90%, 15.81%, 21.21%, and 20.08% were mediated by WBC. In the relationships of 2-OHFlu, 3-OHFlu, 2-OHNa, ∑OHFlu, ∑OHNa, and ∑PAH with FEF_25-75%_, 9.75%, 8.22%, 16.45%, 10.76%, 14.73%, and 14.90% were mediated by WBC (Table 3).

**Table 3:** Mediating effect of WBC in the association between 21 chemicals and lung function indices (*n* = 1,123)

Pathways	Indirect effect (95% CI)	P	Mediated proportion, %	P
**PAHs, Co → WBC → FEV**_**1**_				
2-OHFlu	-29.62 (-56.30, -9.58)	**0.003**	20.67	**0.010**
3-OHFlu	-19.37 (-40.31, -4.36)	**0.006**	17.80	**0.024**
2-OHNa	-37.19 (-68.10, -12.98)	**0.002**	42.50	0.101
∑OHNa	-33.55 (-61.72, -11.33)	**0.001**	45.30	0.157
∑OHFlu	-26.54 (-52.11, -7.55)	**0.003**	16.30	**0.025**
∑PAH	-34.99 (-64.83, -11.63)	**0.001**	38.40	0.099
Co	-30.35 (-63.75, -6.19)	**0.009**	18.54	0.052
**PAHs, Co → WBC → PEF**				
2-OHFlu	-82.07 (-158.36, -25.00)	**0.004**	15.66	**0.006**
3-OHFlu	-54.13 (-114.43, -11.37)	**0.005**	12.77	**0.010**
2-OHNa	-101.14 (-190.87, -33.20)	**0.002**	23.90	**0.015**
∑OHNa	-89.62 (-175.93, -26.04)	**0.003**	21.21	**0.008**
∑OHFlu	-76.40 (-151.04, -21.67)	**0.004**	15.81	**0.012**
∑PAH	-94.36 (-184.80, -27.57)	**0.003**	20.08	**0.007**
Co	-90.28 (-182.35, -20.31)	**0.008**	37.10	0.360
**PAHs, Co → WBC → FEF**_**25-75%**_				
2-OHFlu	-34.40 (-77.10, -2.28)	**0.035**	9.75	**0.035**
3-OHFlu	-23.50 (-56.20, -1.95)	**0.028**	8.22	**0.028**
2-OHNa	-44.09 (-96.03, -5.68)	**0.023**	16.45	**0.032**
∑OHNa	-38.99 (-86.28, -4.20)	**0.024**	14.73	**0.026**
∑OHFlu	-33.36 (-74.94, -3.50)	**0.026**	10.76	**0.028**
∑PAH	-41.24 (-90.89, -4.45)	**0.026**	14.90	**0.028**
Co	-41.72 (-89.08, -6.28)	**0.017**	36.30	0.419

Models were adjusted for gender, age, race, height, PIR, smoking status and drinking status. The urinary concentrations of 11 metals (*n*g/mmol creatine) and 10 PAHs metabolites (*n*g/mmol creatine) were log_10_-transformed. ∑OHFlu, sum of 2-OHFlu, 3-OHFlu, and 9-OHFlu. ∑OHNa, sum of 1-OHNa and 2-OHNa. ∑OHPh, sum of 1-OHPh, 2-OHPh, 3-OHPh, and 4-OHPh. ∑PAH, sum of all the 10 PAHs metabolites

*Abbreviations: 2-OHFlu* 2-Hydroxyfluorene, *3-OHFlu* 3-Hydroxyfluorene, *2-OHNa* 2-Hydroxynapthalene, *Co* cobalt, *FEF*_*25-75%*_ forced expiratory flow between 25% and 75%, *FEV*_*1*_ forced expiratory volume in 1 s, *FVC* forced vital capacity, *PEF* peak expiratory flow

### Sensitivity analysis

In a sensitivity analysis, associations of most of PAH metabolites and metals with lung function were essentially unchanged after excluding participants with COPD or asthma. After excluding participants with COPD, the association of exposures with FEV_1_ persisted except 1-OHPh and the association of exposures with FEV_1_/FVC persisted except 1-OHPh and 2-OHPh. The association of exposures with PEF persisted except Cd, and the association of urinary PAH metabolites with FEF_25-75%_ persisted except 4-OHPh. After excluding participants with asthma, the association of exposures with PEF persisted except 3-OHPh and the association of exposures with FEF_25-75%_ persisted except 4-OHPh (Table S10-S11).

## Discussion

In this study, we assessed the effects of simultaneous exposure to PAHs and metals on lung function and revealed underlying mechanisms. The findings obtained from various statistical analyses consistently indicated that a simultaneous elevation in PAHs and metals was linked to a reduction in FEV_1_, FEV_1_/FVC and FEF_25-75%_, and the mixed effect were dominated by 3-OHFlu, 2-OHPh, and Cd. WBC partly mediated the relationships of 2-OHFlu, 3-OHFlu, and ∑OHFlu with FEV_1_, as well as the relationships of 2-OHFlu, 3-OHFlu, 2-OHNa, ∑OHFlu, ∑OHNa, and ∑PAH with PEF and FEF_25-75%_.

Human beings are subjected to multiple environmental pollutants, and it is meaningful to explore co-exposure effects of various pollutants in epidemiological research [37, 38]. The conventional statistical approaches (such as GLM) are extensively employed to ascertain the associations between exposures and human health owing to their simplicity and ease to explain. However, misleading conclusions may be drawn in the cases of multiple comparisons, multicolinearity, and high dimensionality. In the present study, results from GLM model showed that 10 chemicals, 10 chemicals, 7 chemicals, and 9 chemicals were negatively associated with FEV_1_, FEV_1_/FVC, PEF, and FEF_25-75%_, respectively. Including several chemicals with moderate to high correlations in a multiple linear regression model could induce unreasonable interpretations when unstable and biased standard errors were used to assess the statistical significance of pollutants [39, 40]. LASSO regression analysis is robust for addressing multi-collinearity, reducing dimensionality, and identifying significant components. However, it only fits a linear model, and only one component is chosen from a group of highly correlated pollutants. LASSO regression lacks results that provide information on the effect size, such as the percent change obtained in the other approaches. BKMR is robust to address nonlinearity and interactive effects, as well as overall effects and component selection, which could further capture the association between multiple pollutants and outcomes. Qgcomp allows unbiased inference of multi-pollutant mixing effects and identifies significant contributors to these effects, but data information may be lost during transformation to quantiles [41]. Although these statistical methods had their strengths and limitations (Table S12), they consistently demonstrated that mixtures of PAHs and metals were negatively associated with the FEV_1_/FVC, PEF, and FEF_25-75%_, and identified 3-OHFlu, 2-OHPh, and Cd as the leading stressors for lung function.

There was a negative relationship of PAHs with lung function, which could be substantiated by numerous in vivo and in vitro investigations. A recent study conducted among 45 college students aged 19.9 ± 1.6 years old in China showed that personal exposure to PAHs was negatively associated with FEV_1_, PEF and FEF_25-75%_, but not FVC, which was consistent with our findings [42]. However, our study provided more direct evidence that PAHs and metals were associated with FEV_1_/FVC. NHANES 2009–2012 reported that FEF_25%-75%_, FEV_1_/FVC, and FEV_1_ decreased in participants exposed to high levels of PAHs, which was also similar with our results [43]. Contrary to our findings, other studies have yielded conflicting results. A prior epidemiological investigation carried out in China revealed a notable correlation between exposure to PAHs and the decrease of FEV_1_ and FVC among 3,367 adult participants [44]. Another study among 223 adults aged 38 ± 18 years showed that PAHs exposure was negatively associated with FVC and FEV_1_/FVC, but not FEV_1_, FEF_25%_, or FEF_50%_ [45]. Variations in the study populations, sample size, analysis strategy, and exposure measurements could account for differences in the findings. Findings of many studies could be prone to bias, because they solely focus on exposure to single PAH metabolite. However, in reality, humans are simultaneously exposed to numerous PAHs. Meanwhile, various animal experiments and in vitro cell models have indicated that PAHs exposure could lead to lung inflammation [46, 47], mitochondrial dysfunction [48], and oxidative stress [49], which supported our findings.

There is a growing number of researches concerning adverse effects of metals on lung function. However, only a few studies have assessed the mixture effects of metals on lung function. Our research contributes to this area by presenting a link between metal mixtures and decreased lung function. Specifically, we identified a negative association between Cd and several lung function indices, such as FEV_1_, FEV_1_/FVC, PEF, and FEF_25-75%_. Cd is known as one of the most highly prioritized toxic substances, and its relationship with lung function has been extensively explored in previous research. A recent epidemiological study found a negative association between urinary Cd and FEV_1_% and FVC% among 200 participants recruited from Malaysia [50]. Lampe et al. performed a longitudinal follow-up of 96 men in Boston, Massachusetts, and they found that urinary Cd exposure caused a reduction in FEV_1_ and FEV_1_/FVC [51]. Contrary to our findings, a prospective cohort study conducted among 1,243 coke-oven workers in China revealed a negative relationship of urinary Pb with FEV_1_ and FEV_1_%, but no significant link was found for Cd. This might due to the difference in the exposure level of Cd and Pb [52]. Lung function damage caused by Cd may be due to the release of inflammatory mediators and activation of the immune system after Cd enters the human body. Furthermore, the oxidative stress caused by the accumulation of reactive oxygen species (ROS) may contribute to varying degrees of damage to the human respiratory system caused by cadmium exposure. In our study, adverse effect of Cd exposure does not seem to be mediated by WBC, mediating effects of other inflammatory markers and ROS should also be evaluated in the future study. Our study found that Tl was positively related to PEF. Tl is cumulative and highly toxic, and it could enter the body through inhalation or skin contact. Inconsistent findings were reported in the other three studies. A study among 1,227 American children aged 6–17 years found that there was negative association between urinary Tl and lung health indicators in single-metal models, but not in multiple-metal models [53]. And in two other studies (one conducted among 1,243 workers aged 40.64 ± 7.32 years in Wuhan, China; the other based on 2,363 adults), Tl exposure was suggested to be associated with accelerated decline in lung function [54, 55]. The differences in the results may stem from the following reasons. Firstly, median concentration of urinary Tl was 18.92 (5th, 95th: 8.29, 49.59) ng/mmol creatinine in our study participants, and it was much lower than median concentration reported in the previous studies (median concentrations were 46.00 (31.00, 73.00) and 54.29 ng/mmol creatinine, respectively) [54, 55]. Tl-induced hormesis was largely unknown. Secondly, there were high variability and week repeatability in PEF measurement, and PEF variability is often used to reflect asthma control, not a single-time-point measurement. Lastly, only the positive association between Tl and PEF was observed, not other lung function indices. Further research was warranted to elucidate the relationship between Tl and lung function indices. Exposure level of Co was typically minimal, except during the powder production. Consequently, when examining the connection between Co exposure and lung function, only two groups of researchers have identified a significantly inverse association between Co exposure and FEV_1_ [56, 57].

While the exact mechanism underlying lung function damage caused by PAHs and metals exposure remains uncertain, numerous studies have presented compelling evidences. Prior studies showed that urinary PAH metabolites was positively associated with serum CRP level and total WBC count [58]. Animal studies have also demonstrated that long-term PAHs exposure could induce cytochrome P-450 family enzymes and increase oxidative stress and inflammation in rats [59, 60]. Increase in WBC count indicates the progression of systemic inflammation over time, and previous research has indicated that systemic inflammatory markers may predict an accelerated deterioration of lung function [20–24]. Furthermore, the impact of PAHs on lung function could be attributed to oxidative stress, which stimulates the generation of ROS [61]. After entering and being absorbed into the body, PAHs undergo metabolic transformation facilitated by cytochrome P450 enzymes, resulting in the formation of active semiquinones and subsequent generation of ROS by a free radical intermediate. The excessive activation of ROS could induce oxidative modifications in both DNA and lipids from lung tissue, thereby resulting in the deterioration of lung function [62, 63]. However, there is no data on biomarkers of oxidative stress in this study. Cd caused lung parenchyma damage may be related to disrupting immune response, especially alterations in the mucosal, adaptive, and innate immune responses, thereby enhancing vulnerability to subsequent lung damage and infection risk [64]. Moreover, Cd exposure could induce mitochondrial dysfunction and lead to lipid accumulation in lung cells [65].

Our research has several strengths. To begin with, it offers innovative insights regarding the combined effects of PAHs and metals exposure on lung function in adult. Secondly, mixed exposure models (LASSO, BKMR, and qgcomp) were employed to address different research questions and more robust conclusions were obtained by overcoming the limitations of traditional linear regression model. Furthermore, mediation analysis helps to delve underlying mechanism and offers valuable clues for subsequent research endeavors. Nonetheless, certain limitations need to be acknowledged. Firstly, it is crucial to acknowledge that the present study employed a cross-sectional design, which poses challenges in establishing a conclusive cause-effect association between exposure to PAHs and metals and the deterioration of lung function. Secondly, our findings were based on the United States population and should be cautiously interpreted when extrapolated to other populations. Additionally, the evaluation of PAHs and metals exposure was performed using a spot urine sample, which may not precisely reflect long-term exposure [66]. Levels of PAHs and metals exposure would likely fluctuate within a small range among participants with stable lifestyles and occupations.

Our findings underscore the public health concern regarding pulmonary dysfunction caused by exposure to PAHs and metals. It is crucial to implement effective policies aimed at controlling and minimizing public exposure to PAHs and metals from various sources. Further studies are necessary to validate our research findings and elucidate the underlying biological mechanisms.

## Conclusions

Our research indicates that combined exposure to PAHs and metals is linked to lung function reduction, dominate contributors were 3-OHFlu, 2-OHPh, and Cd, and WBC play a mediating role in the above association. Our findings provide new insights into the negative effects of environmental mixtures on respiratory health and help to enhance public awareness regarding this issue. In the management of environmental pollutants, policy makers should focus on chemicals with highly prioritized toxic and with similar exposure source.

### Supplementary Information

Supplementary Material 1.

Supplementary Material 2.

## Data Availability

All data could be extracted from https://www.cdc.gov/nchs/nhanes/.

## Abbreviations

2-OHFlu: 2-Hydroxyfluorene
3-OHFlu: 3-Hydroxyfluorene
9-OHFlu: 9-Hydroxyfluorene
1-OHNa: 1-Hydroxynapthalene
2-OHNa: 2-Hydroxynapthalene
1-OHPh: 1-Hydroxyphenanthrene
2-OHPh: 2-Hydroxyphenanthrene
3-OHPh: 3-Hydroxyphenanthrene
4-OHPh: 4-Hydroxyphenanthrene
1-OHP: 1-Hydroxypyrene
ATS: American Thoracic Society
Ba: Barium
BKMR: Bayesian kernel machine regression
Cd: Cadmium
*CI*: Confidence interval
Co: Cobalt
Cs: Cesium
DEE: Diesel engine exhaust
FEF_25-75%_: Forced expiratory flow between 25% and 75%
FEV_1_: Forced expiratory volume in 1 s
FVC: Forced vital capacity
GLM: Generalized linear model
LASSO: Least absolute shrinkage and selection operator
LOD: Limits of detection
Mo: Molybdenum
Mn: Manganese
PAH: Polycyclic aromatic hydrocarbons
Pb: Lead
PEF: Peak expiratory flow
PIR: Poverty-income ratio
PIP: Posterior inclusion probabilities
Qgcomp: Quantile-based g-computation
ROS: Reactive oxygen species
Sb: Antimony
SE: Standard error
Tl: Thallium
U: Uranium
W: Tungsten
WBC: White blood cell
