# Supplementary material for: Co-exposure effects of urinary polycyclic aromatic hydrocarbons and metals on lung function: mediating role of systematic inflammation
Source: BMC Pulm Med. 2024 Aug 11;24:386. doi: 10.1186/s12890-024-03173-9 (PMC11316979; doi:10.1186/s12890-024-03173-9)
Supplement: Supplementary file 1 — Supplementary Material 1. [file 12890_2024_3173_MOESM1_ESM.docx]

**Supplemental material**

**Table of Contents**

**Table S1** Distribution of the creatinine-corrected urinary PAHs and metals in NHANES 2011-2012 (*n*=1,123).

**Table S2** Comparisons of baseline characteristics, urinary levels of PAHs metabolites and metals, and lung function level between the whole adult population and selected participants.

**Table S3** Associations of urinary PAHs metabolites and urinary metals with FVC stratified by gender.

**Table S4** Associations of urinary PAHs metabolites and urinary metals with FEV_1_ stratified by gender.

**Table S5** Associations of urinary PAHs metabolites and urinary metals with FEV_1_/FVC (100%) stratified by gender.

**Table S6** Associations of urinary PAHs metabolites and urinary metals with PEF stratified by gender.

**Table S7** Associations of urinary PAHs metabolites and urinary metals with FEF_25-75%_ stratified by gender.

**Table S8** Posterior inclusion probabilities of each exposure for lung function indices using Bayesian kernel machine regression model.

**Table S9** Association between single exposure and WBC using generalized linear model.

**Table 10** Sensitivity analysis for associations of urinary PAHs metabolites and urinary metals with lung function in NHANES 2011-2012 (excluding participants with asthma, *n*=962).

**Table 11** Sensitivity analysis for associations of urinary PAHs metabolites and urinary metals with lung function in NHANES 2011-2012 (excluding participants with COPD, *n*=1,087).

**Table S12** Comparisons of four statistical methods.

**Figure S1** The flowchart of study design.

**Figure S2** Pearson correlations between urinary concentrations of 10 PAH metabolites and 11 metals.

**Figure S3** Univariate exposure-response functions and 95% *CI* for each urinary PAHs and metals.

**Figure S4** The single-exposure effect (estimates and 95% *CI*) for urinary PAHs and metals and lung function.

**Figure S5** Single variable interaction summary for urinary PAHs and metals on lung function.

**Figure S6** Weight of each PAHs and metals in the association with lung function by quantile-based g-computation regression.

| **Table S1** Distribution of the creatinine-corrected urinary PAHs and metals in NHANES 2011-2012 (*n*=1,123). | | | | | | | | |
| --- | --- | --- | --- | --- | --- | --- | --- | --- |
| Exposures | Detection rate | GM | LOD (ng/L) | Percentiles | | | | |
|  |  |  |  | 5^th^ | 25^th^ | 50^th^ | 75^th^ | 95^th^ |
| **Urinary PAHs (ng/mmol creatinine)** | | | | | | | | |
| 2-OHFlu | 100.00% | 32.66 | 10 | 9.43 | 14.66 | 24.03 | 55.97 | 259.87 |
| 3-OHFlu | 98.07% | 12.77 | 10 | 2.74 | 5.31 | 8.91 | 26.04 | 136.26 |
| 9-OHFlu | 99.92% | 34.39 | 10 | 9.11 | 17.97 | 30.97 | 61.43 | 170.31 |
| 1-OHNa | 99.92% | 230.56 | 44 | 38.67 | 74.71 | 185.76 | 591.20 | 2,411.46 |
| 2-OHNa | 100.00% | 551.11 | 42 | 134.00 | 262.68 | 524.37 | 1,129.68 | 2,660.82 |
| 1-OHPh | 99.88% | 17.03 | 10 | 5.86 | 10.16 | 15.74 | 26.56 | 61.40 |
| 2-OHPh | 97.51% | 8.58 | 10 | 2.89 | 5.09 | 7.71 | 13.45 | 33.18 |
| 3-OHPh | 96.71% | 8.39 | 10 | 2.50 | 4.62 | 7.38 | 14.33 | 39.96 |
| 4-OHPh | 76.48% | 2.77 | 10 | 0.75 | 1.54 | 2.51 | 4.46 | 13.26 |
| 1-OHP | 98.83% | 14.33 | 10 | 3.82 | 8.03 | 12.76 | 24.20 | 74.76 |
| **Urinary metals (****ng/mmol creatinine)** | | | | | | | | |
| Ba | 99.28% | 147.86 | 100 | 33.69 | 85.56 | 152.76 | 251.01 | 634.34 |
| Cd | 77.60% | 22.82 | 56 | 5.12 | 12.61 | 22.15 | 41.64 | 99.79 |
| Co | 99.36% | 37.56 | 48 | 15.00 | 23.70 | 35.39 | 54.01 | 124.76 |
| Cs | 100.00% | 495.31 | 120 | 213.68 | 349.53 | 487.02 | 703.55 | 1,155.51 |
| Mo | 100.00% | 4,239.03 | 80 | 1,554.21 | 2,854.43 | 4,264.60 | 6,267.63 | 1,1781.25 |
| Mn | 69.13% | 15.55 | 990 | 3.98 | 9.08 | 15.14 | 26.61 | 61.69 |
| Pb | 96.01% | 46.00 | 80 | 14.14 | 28.83 | 45.35 | 73.83 | 164.86 |
| Sb | 86.10% | 6.55 | 41 | 2.26 | 3.95 | 6.33 | 10.07 | 24.36 |
| Tl | 99.44% | 19.17 | 20 | 8.29 | 13.43 | 18.92 | 26.45 | 49.59 |
| W | 86.47% | 8.21 | 26 | 2.54 | 4.74 | 7.79 | 13.49 | 37.54 |
| U | 73.84% | 0.73 | 3.3 | 0.22 | 0.41 | 0.69 | 1.15 | 3.22 |
| **Abbreviations:** PAHs, polycyclic aromatic hydrocarbons; GM, geometric mean; LOD, limits of detection. | | | | | | | | |

| **Table S2.** Comparisons of baseline characteristics, urinary levels of PAHs metabolites and metals, and lung function level between the whole adult population and selected participants. | | | | | |
| --- | --- | --- | --- | --- | --- |
| Variables | All adults | |  | Study participants | |
|  | *n* | Values |  | *n* | Values |
| **General characteristics** | | | | |  |
| Age (years) | 5,560 | 47.37±0.38 |  | 1,123 | 44.93±1.02 |
| Gender | | | | | |
| Male | 2,740 | 50.60% |  | 595 | 50.50% |
| Female | 2,820 | 49.40% |  | 528 | 49.50% |
| Race |  |  |  |  |  |
| Mexican American | 540 | 7.70 % |  | 98 | 6.30% |
| Other Hispanic | 578 | 6.60% |  | 115 | 6.20% |
| Non-Hispanic White | 2,041 | 66.50% |  | 422 | 69.50% |
| Non-Hispanic Black | 1,455 | 11.50% |  | 295 | 10.60% |
| Others | 946 | 7.70% |  | 193 | 7.40% |
| Height (cm) | 5,249 | 168.59±0.23 |  | 1,123 | 169.33±0.38 |
| Cigarette smoking | | | | | |
| Never | 3,184 | 56.30% |  | 646 | 56.60% |
| Ever | 2,369 | 43.70% |  | 477 | 43.40% |
| Alcohol drinking |  |  |  |  |  |
| Never | 3,413 | 79.90% |  | 861 | 83.8% |
| Ever | 1,275 | 20.10% |  | 262 | 16.20% |
| Ratio of family income to poverty | | | | |  |
| 0-4.99 | 3,536 | 75.70% |  | 912 | 76.90% |
| ≥5 | 737 | 24.30% |  | 211 | 23.10% |
| **PAH metabolites** (*n*g/mmol creatinine) | | | | |  |
| 2-OHFlu | 1,705 | 24.68 (9.60, 249.90) |  | 1,123 | 24.03 (9.43, 259.87) |
| 3-OHFlu | 1,701 | 8.99 (2.74, 132.00) |  | 1,123 | 8.92 (2.74, 136.26) |
| 9-OHFlu | 1,704 | 30.91 (9.08, 163.42) |  | 1,123 | 30.97 (9.11, 170.31) |
| 1-OHNa | 1,705 | 190.84 (37.76, 2,536.27) |  | 1,123 | 185.76 (38.67, 2,411.46) |
| 2-OHNa | 1,705 | 586.74 (141.89, 2,745.85) |  | 1,123 | 524.37 (134.00, 2,660.82) |
| 1-OHPh | 1,705 | 15.36 (5.30, 57.05) |  | 1,123 | 15.74 (5.86, 61.40) |
| 2-OHPh | 1,702 | 7.69 (2.80, 28.13) |  | 1,123 | 7.71 (2.89, 33.18) |
| 3-OHPh | 1,704 | 7.49 (2.41, 36.18) |  | 1,123 | 7.38 (2.50, 39.96) |
| 4-OHPh | 1,700 | 2.54 (0.78, 11.46) |  | 1,123 | 2.51 (0.75, 13.26) |
| 1-OHP | 1,702 | 13.10 (3.41, 61.01) |  | 1,123 | 12.76 (3.82, 74.76) |
| **Urinary metals** (*n*g/mmol creatinine) | | | | |  |
| Ba | 1,669 | 138.59 (27.78, 619.15) |  | 1,123 | 152.76 (33.69, 634.34) |
| Cd | 1,669 | 30.22 (6.56, 129.96) |  | 1,123 | 22.15 (5.12, 99.79) |
| Co | 1,669 | 37.70 (14.10, 154.11) |  | 1,123 | 35.39 (15.00, 124.76) |
| Cs | 1,668 | 481.63 (215.74, 1,167.41) |  | 1,123 | 487.02 (213.68, 1,155.51) |
| Mo | 1,669 | 4,463.68 (1,591.76, 14,492.67) |  | 1,123 | 4,264.60 (1,554.21, 11,781.25) |
| Mn | 1,669 | 15.40 (4.08, 64.61) |  | 1,123 | 15.13 (3.98, 61.69) |
| Pb | 1,669 | 48.47 (15.49, 177.04) |  | 1,123 | 45.35 (14.14, 164.86) |
| Sb | 1,669 | 6.32 (2.44, 23.43) |  | 1,123 | 6.33 (2.26, 24.36) |
| Tl | 1,669 | 18.67 (7.53, 52.70) |  | 1,123 | 18.92 (8.29, 49.59) |
| W | 1,660 | 8.00 (2.58, 41.66) |  | 1,123 | 7.79 (2.54, 37.54) |
| U | 1,669 | 0.72 (0.22,3.55) |  | 1,123 | 0.69 (0.22, 3.22) |
| **Lung function indices** | | | | |  |
| FVC (mL) | 4,212 | 4,098.67±29.61 |  | 1,123 | 4,132.66±42.48 |
| FEV_1_ (mL) | 4,230 | 3,199.00±29.20 |  | 1,123 | 3,221.70±37.84 |
| FEV_1_/FVC (100%) | 4,190 | 78.03±0.35 |  | 1,123 | 78.05±0.44 |
| PEF (mL/s) | 4,340 | 8,302.25±84.78 |  | 1,123 | 8,445.63±118.37 |
| FEF_25-75%_ (mL/s) | 4,340 | 3,007.18±46.30 |  | 1,123 | 3,005.46±62.77 |
| **Note:** Continuous variables were presented as mean ± SE or median (5th, 95th percentiles). Categorical variables were presented as *n* (%). | | | | | |

| Table S3 Associations of urinary PAHs metabolites and urinary metals with FVC stratified by gender. | | | | | | |
| --- | --- | --- | --- | --- | --- | --- |
| Exposures | Male | |  | Female | | *P*_interaction_ |
|  | *β* (95% *CI*) | *P* |  | *β* (95% *CI*) | *P* |  |
| **Urinary PAHs** (*n*g/mmol creatinine) | | | | | | |
| 2-OHFlu | -29.60 (-221.37, 162.17) | 0.749 |  | -37.02 (-173.09, 99.05) | 0.574 | 0.977 |
| 3-OHFlu | -8.41 (-154.11, 137.30) | 0.905 |  | -20.81 (-124.31, 82.69) | 0.677 | 0.915 |
| 9-OHFlu | -147.46 (-410.24, 115.33) | 0.253 |  | -95.26 (-227.49, 36.97) | 0.147 | 0.728 |
| 1-OHNa | 13.82 (-127.84, 155.48) | 0.839 |  | 32.67 (-95.80, 161.14) | 0.600 | 0.683 |
| 2-OHNa | 32.35 (-147.09, 211.79) | 0.708 |  | 21.67 (-108.47, 151.82) | 0.730 | 0.971 |
| 1-OHPh | -85.50 (-311.66, 140.65) | 0.436 |  | -76.07 (-213.95, 61.82) | 0.261 | 0.929 |
| 2-OHPh | -127.48 (-401.39, 146.43) | 0.340 |  | -115.85 (-232.48, 0.780) | 0.051 | 0.933 |
| 3-OHPh | -60.44 (-275.59, 154.70) | 0.561 |  | -39.36 (-183.59, 104.86) | 0.572 | 0.890 |
| 4-OHPh | -115.86 (-369.97, 138.26) | 0.350 |  | -88.21 (-167.83, -8.59) | **0.032** | 0.828 |
| 1-OHP | -53.16 (-262.44, 156.12) | 0.599 |  | -92.45 (-253.16, 68.27) | 0.242 | 0.584 |
| ∑OHFlu | -86.98 (-305.87, 131.92) | 0.414 |  | -72.77 (-217.82, 72.28) | 0.305 | 0.903 |
| ∑OHNa | 14.33 (-176.63, 205.29) | 0.876 |  | 31.34 (-159.82, 222.50) | 0.734 | 0.792 |
| ∑OHPh | -86.59 (-328.85, 155.66) | 0.461 |  | -72.22 (-212.80, 68.37) | 0.294 | 0.993 |
| ∑PAH | -9.21 (-210.21, 191.79) | 0.924 |  | 17.20 (-192.06, 226.45) | 0.864 | 0.767 |
| **Urinary metals** (*n*g/mmol creatinine) | | | | | |  |
| Ba | -246.73 (-475.76, -17.70) | **0.036** |  | 1.24 (-206.06, 208.53) | 0.990 | 0.214 |
| Cd | -6.19 (-235.78, 223.39) | 0.955 |  | -69.42 (-215.54, 76.69) | 0.330 | 0.646 |
| Co | -388.25 (-757.18, -19.32) | **0.040** |  | -4.96 (-171.94, 162.03) | 0.951 | **0.033** |
| Cs | -3.18 (-345.45, 339.09) | 0.985 |  | 12.99 (-159.80, 185.79) | 0.876 | 0.776 |
| Mo | -355.17 (-666.57, -43.77) | **0.028** |  | -76.56 (-239.83, 86.70) | 0.336 | 0.123 |
| Mn | -232.44 (-496.75, 31.87) | 0.081 |  | 60.77 (-52.15, 173.70) | 0.272 | 0.066 |
| Pb | 33.11 (-276.96, 343.18) | 0.824 |  | 125.21 (-49.95, 300.37) | 0.150 | 0.341 |
| Sb | -25.42 (-245.75, 194.92) | 0.811 |  | -103.64 (-281.37, 74.08) | 0.235 | 0.449 |
| Tl | 84.30 (-373.75, 542.35) | 0.703 |  | 8.99 (-189.22, 207.20) | 0.925 | 0.598 |
| W | -133.87 (-396.56, 128.82) | 0.297 |  | -66.15 (-196.76, 64.47) | 0.300 | 0.784 |
| U | -126.53 (-402.05, 148.99) | 0.346 |  | -119.81 (-228.26, -11.37) | **0.032** | 0.880 |
| **Notes:** Log_10_-transformed, creatinine-corrected urinary concentration of each PAHs metabolite and metal was modeled as continuous variables to calculate coefficient and 95% confidence intervals (*CI*) for FVC. Generalized linear model was adjusted for age, race, height, family poverty-income ratio, smoking and drinking status. ∑OHNa, sum of 1-OHNa and 2-OHNa. ∑OHFlu, sum of 2-OHFlu, 3-OHFlu, and 9-OHFlu. ∑OHNa, sum of 1-OHNa and 2-OHNa. ∑OHPh, sum of 1-OHPh, 2-OHPh, 3-OHPh, and 4-OHPh. ∑PAH, sum of all the 10 PAHs metabolites. | | | | | | |

| Table S4 Associations of urinary PAHs metabolites and urinary metals with FEV_1_ stratified by gender. | | | | | | |
| --- | --- | --- | --- | --- | --- | --- |
| Exposures | Male | |  | Female | | *P*_interaction_ |
|  | *β* (95% *CI*) | *P* |  | *β* (95% *CI*) | *P* |  |
| **Urinary PAHs** (*n*g/mmol creatinine) | | | | | | |
| 2-OHFlu | -171.86 (-335.44, -8.28) | **0.041** |  | -147.46 (-248.77, -46.15) | **0.007** | 0.549 |
| 3-OHFlu | -130.23 (-246.41, -14.05) | **0.030** |  | -122.89 (-188.77, -57.00) | **0.001** | 0.645 |
| 9-OHFlu | -153.25 (-396.38, 89.88) | 0.201 |  | -157.89 (-236.08, -79.70) | **<0.001** | 0.804 |
| 1-OHNa | -93.62 (-223.18, 35.95) | 0.146 |  | -25.71 (-122.33, 70.92) | 0.582 | 0.133 |
| 2-OHNa | -134.24 (-318.41, 49.94) | 0.143 |  | -82.29 (-199.63, 35.04) | 0.157 | 0.502 |
| 1-OHPh | -154.99 (-351.66, 41.68) | 0.115 |  | -96.43 (-209.27, 16.41) | 0.089 | 0.502 |
| 2-OHPh | -191.56 (-429.62, 46.49) | 0.108 |  | -176.03 (-304.65, -47.40) | **0.010** | 0.781 |
| 3-OHPh | -148.71 (-323.95, 26.52) | 0.091 |  | -132.66 (-260.75, -4.57) | **0.043** | 0.744 |
| 4-OHPh | -170.21 (-399.25, 58.84) | 0.135 |  | -156.70 (-252.76, -60.65) | **0.003** | 0.734 |
| 1-OHP | -134.09 (-299.78, 31.60) | 0.106 |  | -161.32 (-289.37, -33.27) | **0.017** | 0.645 |
| ∑OHFlu | -189.27 (-385.58, 7.04) | 0.058 |  | -167.91 (-260.37, -75.46) | **0.001** | 0.599 |
| ∑OHNa | -145.65 (-327.35, 36.05) | 0.109 |  | -58.76 (-204.39, 86.86) | 0.406 | 0.303 |
| ∑OHPh | -170.51 (-380.58, 39.57) | 0.105 |  | -131.39 (-253.42, -9.36) | **0.036** | 0.620 |
| ∑PAH | -166.12 (-359.07, 26.84) | 0.087 |  | -73.12 (-229.87, 83.63) | 0.339 | 0.307 |
| **Urinary metals** (*n*g/mmol creatinine) | | | | | | |
| Ba | -229.86 (-366.17, -93.56) | **0.002** |  | 37.73 (-153.19, 228.65) | 0.682 | 0.062 |
| Cd | -231.29 (-437.97, -24.61) | **0.030** |  | -146.10 (-284.57, -7.63) | **0.040** | 0.110 |
| Co | -310.45 (-607.42, -13.48) | **0.042** |  | 20.94 (-134.70, 176.58) | 0.780 | **0.027** |
| Cs | 53.89 (-239.85, 347.64) | 0.704 |  | 0.730 (-174.12, 175.58) | 0.993 | 0.841 |
| Mo | -113.77 (-434.34, 206.79) | 0.464 |  | 19.92 (-100.88, 140.72) | 0.732 | 0.431 |
| Mn | -132.19 (-343.23, 78.85) | 0.204 |  | 55.18 (-36.42, 146.78) | 0.221 | 0.085 |
| Pb | -56.98 (-368.44, 254.49) | 0.704 |  | 45.12 (-119.76, 210.00) | 0.571 | 0.262 |
| Sb | 0.25 (-137.98, 138.49) | 0.997 |  | -70.26 (-240.47, 99.95) | 0.396 | 0.502 |
| Tl | 121.07 (-177.88, 420.02) | 0.405 |  | 54.54 (-136.08, 245.16) | 0.554 | 0.575 |
| W | -50.88 (-428.24, 100.98) | 0.696 |  | -42.30 (-154.48, 69.89) | 0.437 | 0.902 |
| U | -210.14 (-415.63, -4.66) | **0.046** |  | -87.51 (-196.71, 21.68) | 0.109 | 0.279 |
| **Note:** Log_10_-transformed, creatinine-corrected urinary concentration of each PAHs metabolite and metal was modeled as continuous variables to calculate coefficient and 95% confidence intervals (*CI*) for FEV_1_. Generalized linear model was adjusted for age, race, height, family poverty-income ratio, smoking and drinking status. ∑OHNa, sum of 1-OHNa and 2-OHNa. ∑OHFlu, sum of 2-OHFlu, 3-OHFlu, and 9-OHFlu. ∑OHNa, sum of 1-OHNa and 2-OHNa. ∑OHPh, sum of 1-OHPh, 2-OHPh, 3-OHPh, and 4-OHPh. ∑PAH, sum of all the 10 PAHs metabolites. | | | | | | |

| Table S5 Associations of urinary PAHs metabolites and urinary metals with FEV_1_/FVC (100%) stratified by gender. | | | | | | |
| --- | --- | --- | --- | --- | --- | --- |
| Exposures | Male | |  | Female | | *P*_interaction_ |
|  | *β* (95% *CI*) | *P* |  | *β* (95% *CI*) | *P* |  |
| **Urinary PAHs** (*n*g/mmol creatinine) | | | | | | |
| 2-OHFlu | -3.12 (-4.93, -1.30) | **0.002** |  | -3.66 (-5.93, -1.39) | **0.003** | 0.790 |
| 3-OHFlu | -2.57 (-4.21, -0.93) | **0.004** |  | -3.31 (-4.99, -1.62) | **<0.001** | 0.579 |
| 9-OHFlu | -0.83 (-3.26,1.62) | 0.485 |  | -2.48 (-4.65, -0.31) | **0.028** | 0.236 |
| 1-OHNa | -2.16 (-3.37, -0.95) | **0.002** |  | -1.58 (-2.62, -0.54) | **0.005** | 0.401 |
| 2-OHNa | -3.41 (-5.33, -1.48) | **0.002** |  | -3.03 (-5.75, -0.32) | **0.031** | 0.618 |
| 1-OHPh | -1.63 (-4.06,0.80) | 0.176 |  | -0.86 (-3.07, 1.35) | 0.424 | 0.603 |
| 2-OHPh | -1.71 (-3.64, 0.21) | 0.077 |  | -2.38 (-5.13, 0.37) | 0.086 | 0.586 |
| 3-OHPh | -1.85 (-3.93, 0.22) | 0.077 |  | -3.01 (-5.44, -0.58) | **0.018** | 0.406 |
| 4-OHPh | -1.45 (-3.78, 0.88) | 0.206 |  | -2.38 (-5.38, 0.62) | 0.113 | 0.561 |
| 1-OHP | -1.85 (-3.53, -0.18) | **0.032** |  | -2.56 (-5.05, -0.08) | **0.044** | 0.466 |
| ∑OHFlu | -2.48 (-4.45, -0.52) | **0.016** |  | -3.41 (-5.64, -1.17) | **0.005** | 0.500 |
| ∑OHNa | -3.31 (-4.88, -1.74) | **<0.001** |  | -2.48 (-4.03, -0.93) | **0.004** | 0.338 |
| ∑OHPh | -1.89 (-4.31, 0.53) | 0.118 |  | -2.06 (-4.64, 0.52) | 0.111 | 0.849 |
| ∑PAH | -3.34 (-5.07, -1.61) | **<0.001** |  | -2.57 (-4.17, -0.96) | **0.004** | 0.371 |
| **Urinary metals** (*n*g/mmol creatinine) | | | | | | |
| Ba | -0.75 (-2.76, 1.26) | 0.443 |  | 1.57 (-1.24, 4.37) | 0.255 | 0.237 |
| Cd | -4.90 (-8.16, -1.65) | **0.006** |  | -3.30 (-5.20, -1.40) | **0.002** | 0.553 |
| Co | -1.46 (-6.46, 3.53) | 0.546 |  | 1.10 (-1.27, 3.48) | 0.341 | 0.407 |
| Cs | 0.98 (-2.19, 4.15) | 0.523 |  | -0.01 (-2.69, 2.67) | 0.992 | 0.508 |
| Mo | 3.22 (-0.77, 7.21) | 0.107 |  | 2.57 (0.04, 5.10) | **0.047** | 0.601 |
| Mn | 0.75 (-1.96, 3.45) | 0.567 |  | 0.37 (-1.93, 2.66) | 0.740 | 0.740 |
| Pb | -2.05 (-4.51, 0.41) | 0.096 |  | -1.56 (-3.58, 0.47) | 0.123 | 0.936 |
| Sb | 0.45 (-1.12, 2.02) | 0.557 |  | -0.08 (-3.14, 2.97) | 0.955 | 0.784 |
| Tl | 1.58 (-1.95, 5.10) | 0.358 |  | 1.97 (-1.04, 4.97) | 0.185 | 0.841 |
| W | 0.76 (-1.23, 2.75) | 0.432 |  | 0.12 (-1.92, 2.16) | 0.902 | 0.691 |
| U | -2.39 (-5.80, 1.01) | 0.157 |  | 0.082 (-1.84, 02.01) | 0.930 | 0.223 |
| **Note:** Log_10_-transformed, creatinine-corrected urinary concentration of each PAHs metabolite and metal was modeled as continuous variables to calculate coefficient and 95% confidence intervals (*CI*) for FEV_1_/FVC (100%). Generalized linear model was adjusted for age, race, height, family poverty-income ratio, smoking and drinking status. ∑OHFlu, sum of 2-OHFlu, 3-OHFlu, and 9-OHFlu. ∑OHNa, sum of 1-OHNa and 2-OHNa. ∑OHPh, sum of 1-OHPh, 2-OHPh, 3-OHPh, and 4-OHPh. ∑PAH, sum of all the 10 PAHs metabolites. | | | | | | |

| Table S6 Associations of urinary PAHs metabolites and urinary metals with PEF stratified by gender. | | | | | | |
| --- | --- | --- | --- | --- | --- | --- |
| Exposures | Male | |  | Female | | *P*_interaction_ |
|  | *β* (95% *CI*) | *P* |  | *β* (95% *CI*) | *P* |  |
| **Urinary PAHs** (*n*g/mmol creatinine) | | | | | | |
| 2-OHFlu | -700.10 (-1143.47, -256.72) | **0.004** |  | -326.83 (-776.39, 122.74) | 0.144 | 0.213 |
| 3-OHFlu | -606.92 (-1010.70, -203.13) | **0.006** |  | -228.35 (-568.11, 111.40) | 0.174 | 0.142 |
| 9-OHFlu | -549.85 (-1246.77, 147.07) | 0.114 |  | -125.65 (-451.23, 199.93) | 0.427 | 0.145 |
| 1-OHNa | -409.40 (-785.53, -33.27) | **0.035** |  | -221.33 (-443.80, 1.14) | 0.051 | 0.291 |
| 2-OHNa | -642.75 (-1198.58, -86.91) | **0.026** |  | -295.04 (-683.96, 93.87) | 0.128 | 0.212 |
| 1-OHPh | -520.72 (-1065.93, 24.49) | 0.060 |  | 207.35 (-234.50, 649.20) | 0.336 | **0.040** |
| 2-OHPh | -637.80 (-1281.03, 5.43) | 0.052 |  | -76.60 (-652.79, 499.59) | 0.783 | 0.207 |
| 3-OHPh | -619.80 (-1125.05, -114.54) | **0.019** |  | -136.11 (-604.68, 332.46) | 0.548 | 0.155 |
| 4-OHPh | -478.15 (-1166.40, 210.10) | 0.161 |  | 136.24 (-401.47, 673.96) | 0.600 | 0.163 |
| 1-OHP | -456.37 (-833.52, -79.22) | **0.021** |  | -206.93 (-665.25, 251.39) | 0.354 | 0.568 |
| ∑OHFlu | -747.14 (-1278.42, -215.86) | **0.009** |  | -204.15 (-629.30, 221.00) | 0.325 | **0.042** |
| ∑OHNa | -607.86 (-1094.22, -121.50) | **0.017** |  | -332.30 (-632.39, -32.20) | **0.032** | 0.294 |
| ∑OHPh | -628.84 (-1204.61, -53.08) | **0.034** |  | 101.10 (-393.97, 596.16) | 0.672 | **0.050** |
| ∑PAH | -668.68 (-1169.45, -167.90) | **0.012** |  | -333.21 (-655.69, -10.74) | **0.044** | 0.208 |
| **Urinary metals** (*n*g/mmol creatinine) | | | | | | |
| Ba | -544.52 (-1038.23, -50.80) | **0.033** |  | 169.64 (-231.82, 571.10) | 0.385 | **0.008** |
| Cd | -716.39 (-1227.11, -205.68) | **0.009** |  | -100.51 (-623.80, 422.77) | 0.690 | 0.158 |
| Co | -486.99 (-1561.55, 587.58) | 0.352 |  | 51.62 (-386.36, 489.60) | 0.807 | 0.284 |
| Cs | 73.56 (-735.62, 882.73) | 0.850 |  | 472.38 (-70.04, 1014.81) | 0.084 | 0.470 |
| Mo | -32.83 (-1116.48, 1050.82) | 0.950 |  | 142.49 (-357.85, 642.83) | 0.556 | 0.922 |
| Mn | 127.15 (-555.18, 809.48) | 0.699 |  | 366.00 (-122.42, 854.42) | 0.132 | 0.483 |
| Pb | -479.71 (-1155.14, 195.72) | 0.152 |  | 6.70 (-561.83, 575.22) | 0.981 | 0.284 |
| Sb | 156.56 (-143.43, 456.56) | 0.286 |  | 103.45 (-504.81, 711.70) | 0.724 | 0.777 |
| Tl | 566.48 (-414.71, 1547.67) | 0.240 |  | 902.39 (367.77, 1437.02) | **0.002** | 0.578 |
| W | -372.04 (-1069.26, 325.18) | 0.276 |  | -33.73 (-467.93, 400.46) | 0.872 | 0.521 |
| U | -776.31 (-1419.43, -133.19) | **0.021** |  | 172.55 (-347.00, 692.09) | 0.493 | **0.044** |
| **Note:** Log_10_-transformed, creatinine-corrected urinary concentration of each PAHs metabolite and metal was modeled as continuous variables to calculate coefficient and 95% confidence intervals (*CI*) for PEF. Generalized linear model was adjusted for age, race, height, family poverty-income ratio, smoking and drinking status. ∑OHFlu, sum of 2-OHFlu, 3-OHFlu, and 9-OHFlu. ∑OHNa, sum of 1-OHNa and 2-OHNa. ∑OHPh, sum of 1-OHPh, 2-OHPh, 3-OHPh, and 4-OHPh. ∑PAH, sum of all the 10 PAHs metabolites. | | | | | | |

| Table S7 Associations of urinary PAHs metabolites and urinary metals with FEF_25-75%_ stratified by gender. | | | | | | |
| --- | --- | --- | --- | --- | --- | --- |
| Exposures | Male | |  | Female | | *P*_interaction_ |
|  | *β* (95% *CI*) | *P* |  | *β* (95% *CI*) | *P* |  |
| **Urinary PAHs** (*n*g/mmol creatinine) | | | | | | |
| 2-OHFlu | -326.15 (-593.91, -58.39) | **0.020** |  | -410.32 (-586.49, -234.15) | **<0.001** | 0.959 |
| 3-OHFlu | -247.63 (-443.18, -52.08) | **0.016** |  | -368.03 (-485.68, -250.38) | **<0.001** | 0.661 |
| 9-OHFlu | -102.46 (-541.13, 336.20) | 0.629 |  | -285.74 (-488.30, -83.18) | **0.009** | 0.658 |
| 1-OHNa | -253.74 (-487.52, -19.97) | **0.035** |  | -149.14 (-276.33, -21.95) | **0.024** | 0.071 |
| 2-OHNa | -316.12 (-614.08, -18.15) | **0.039** |  | -275.40 (-566.97, 16.18) | 0.063 | 0.619 |
| 1-OHPh | -185.98 (-542.36, 170.40) | 0.286 |  | -129.49 (-363.33, 104.35) | 0.259 | 0.577 |
| 2-OHPh | -228.30 (-551.70, 95.09) | 0.155 |  | -379.87 (-642.07, -117.67) | **0.007** | 0.605 |
| 3-OHPh | -224.80 (-510.26, 60.66) | 0.115 |  | -343.67 (-611.31, -76.04) | **0.015** | 0.739 |
| 4-OHPh | -219.27 (-607.41, 168.87) | 0.250 |  | -264.37 (-519.00, -9.75) | **0.043** | 0.934 |
| 1-OHP | -183.08 (-421.99, 55.82) | 0.124 |  | -340.74 (-573.16, -108.32) | **0.007** | 0.208 |
| ∑OHFlu | -268.84 (-623.79, 86.11) | 0.129 |  | -386.95 (-571.82, -202.09) | **<0.001** | 0.909 |
| ∑OHNa | -360.56 (-658.11, -63.02) | **0.020** |  | -237.06 (-453.29, -20.82) | **0.034** | 0.221 |
| ∑OHPh | -229.00 (-580.51, 122.51) | 0.187 |  | -246.80 (-503.90, 10.30) | 0.059 | 0.831 |
| ∑PAH | -367.75 (-697.88, -37.62) | **0.031** |  | -252.68 (-471.23, -34.13) | **0.026** | 0.250 |
| **Urinary metals** (*n*g/mmol creatinine) | | | | | | |
| Ba | -188.55 (-372.87, -4.22) | **0.046** |  | 122.65 (-190.97, 436.27) | 0.421 | 0.184 |
| Cd | -447.70 (-853.73, -41.67) | **0.033** |  | -354.17 (-595.82, -112.52) | **0.007** | 0.109 |
| Co | -135.70 (-726.36, 454.96) | 0.634 |  | 48.05 (-357.90, 453.99) | 0.806 | 0.564 |
| Cs | 188.04 (-332.73, 708.81) | 0.457 |  | 31.62 (-412.96, 476.20) | 0.883 | 0.993 |
| Mo | 285.32 (-349.36, 920.00) | 0.356 |  | 152.23 (-139.38, 443.84) | 0.286 | 0.515 |
| Mn | -20.64 (-412.45, 371.17) | 0.913 |  | 78.49 (-107.53, 264.52) | 0.386 | 0.496 |
| Pb | -115.21 (-625.94, 395.52) | 0.640 |  | -128.84 (-495.44, 237.76) | 0.469 | 0.553 |
| Sb | 151.70 (-74.91, 378.32) | 0.176 |  | -20.06 (-428.62, 388.49) | 0.919 | 0.484 |
| Tl | 283.00 (-129.4, 695.40) | 0.166 |  | 257.42 (-226.20, 741.05) | 0.277 | 0.825 |
| W | 94.25 (-317.01, 505.50) | 0.635 |  | -58.99 (-373.66, 255.67) | 0.697 | 0.404 |
| U | -204.49 (-583.83, 174.86) | 0.271 |  | -34.84 (-292.42, 222.74) | 0.779 | 0.368 |
| **Note:** Log_10_-transformed, creatinine-corrected urinary concentration of each PAHs metabolite and metal was modeled as continuous variables to calculate coefficient and 95% confidence intervals (*CI*) for FEF_25-75%_. Generalized linear model was adjusted for age, race, height, family poverty-income ratio, smoking and drinking status. ∑OHFlu, sum of 2-OHFlu, 3-OHFlu, and 9-OHFlu. ∑OHNa, sum of 1-OHNa and 2-OHNa. ∑OHPh, sum of 1-OHPh, 2-OHPh, 3-OHPh, and 4-OHPh. ∑PAH, sum of all the 10 PAHs metabolites. | | | | | | |

| **Table S8** Posterior inclusion probabilities of each exposure for lung function indices using Bayesian kernel machine regression model. | | | | | |
| --- | --- | --- | --- | --- | --- |
| Exposures | PIPs | | | | |
|  | FVC | FEV_1_ | FEV_1_/FVC | PEF | FEF_25-75%_ |
| **Urinary PAHs** (*n*g/mmol creatinine) | | | | | |
| 2-OHFlu | 0.140 | **0.794** | 0.076 | **0.963** | 0.273 |
| 3-OHFlu | 0.041 | 0.468 | **0.947** | 0.336 | **0.813** |
| 9-OHFlu | 0.134 | **0.593** | 0.004 | 0.368 | 0.074 |
| 1-OHNa | 0.043 | 0.425 | 0.0003 | 0.217 | 0.073 |
| 2-OHNa | 0.024 | 0.369 | 0.002 | 0.240 | 0.082 |
| 1-OHPh | **0.854** | **0.975** | 0.035 | **0.903** | 0.293 |
| 2-OHPh | **0.835** | **0.783** | 0.025 | 0.322 | 0.093 |
| 3-OHPh | 0.077 | 0.469 | 0.066 | 0.273 | 0.149 |
| 4-OHPh | 0.097 | 0.500 | 0.002 | 0.338 | 0.080 |
| 1-OHP | 0.054 | 0.461 | 0.098 | 0.320 | 0.286 |
| **Urinary metals** (*n*g/mmol creatinine) | | | | | |
| Ba | 0.036 | 0.349 | 0.005 | 0.415 | 0.106 |
| Cd | 0.058 | **0.687** | **0.987** | 0.350 | **0.590** |
| Co | 0.100 | 0.437 | 0.004 | 0.299 | 0.081 |
| Cs | **0.948** | **0.856** | 0.010 | 0.398 | 0.170 |
| Mo | 0.092 | 0.418 | 0.012 | 0.273 | 0.096 |
| Mn | 0.067 | 0.427 | 0.007 | 0.283 | 0.153 |
| Pb | 0.049 | 0.485 | 0.007 | 0.347 | 0.095 |
| Sb | 0.031 | 0.319 | 0.004 | 0.282 | 0.194 |
| Tl | 0.176 | **0.661** | 0.012 | **0.585** | 0.075 |
| W | 0.058 | 0.382 | 0.003 | 0.354 | 0.088 |
| U | 0.103 | 0.338 | 0.001 | 0.289 | 0.072 |
| **Notes:** Bayesian kernel machine regression model was adjusted for gender, age, race, height, family poverty-income ratio, smoking status and drinking status. | | | | | |
|  | | | | | |

| **Table S9** Association between single exposure and WBC using generalized linear model. | | |
| --- | --- | --- |
| Exposures | WBC | |
|  | *β* (95% *CI*) | *P* |
| **Urinary PAHs** (*n*g/mmol creatinine) | | |
| 2-OHFlu | 0.85 (0.37, 1.33) | **0.002** |
| 3-OHFlu | 0.53 (0.08, 0.99) | **0.025** |
| 9-OHFlu | 0.57 ( -0.01, 1.15) | 0.054 |
| 1-OHNa | 0.33 (-0.16, 0.81) | 0.174 |
| 2-OHNa | 0.99 (0.38, 1.60) | **0.003** |
| 1-OHPh | 0.26 (-0.30, 0.82) | 0.348 |
| 2-OHPh | 0.70 (-0.05, 1.46) | 0.066 |
| 3-OHPh | 0.49 (-0.13, 1.12) | 0.114 |
| 4-OHPh | 0.49 (-0.09, 1.08) | 0.091 |
| 1-OHP | 0.52 ( -0.12, 1.15) | 0.107 |
| ∑OHFlu | 0.76 (0.18, 1.33) | **0.013** |
| ∑OHNa | 0.88 (0.15, 1.61) | **0.022** |
| ∑OHPh | 0.48 (-0.20, 1.16) | 0.157 |
| ∑PAH | 0.93 (0.16, 1.69) | **0.021** |
| **Urinary Metals** (*n*g/mmol creatinine) | | |
| Ba | -0.02 (-0.37, 0.33) | 0.913 |
| Cd | 0.05 (-0.41, 0.50) | 0.825 |
| Co | 0.76 (0.12, 1.40) | **0.020** |
| Cs | -0.29 (-0.98, 0.40) | 0.382 |
| Mo | -0.19 (-0.79, 0.41) | 0.511 |
| Mn | -0.24 (-0.72, 0.25) | 0.319 |
| Pb | -0.13 (-0.77, 0.52) | 0.683 |
| Sb | 0.24 (-0.25, 0.73) | 0.319 |
| Tl | -0.58 (-1.40, 0.24) | 0.152 |
| W | 0.28 (-0.15, 0.72) | 0.189 |
| U | 0.05 (-0.28, 0.39) | 0.745 |
| **Notes:** Generalized linear model was adjusted for gender, age, race, height, family poverty-income ratio, smoking status and drinking status. ∑OHFlu, sum of 2-OHFlu, 3-OHFlu, and 9-OHFlu. ∑OHNa, sum of 1-OHNa and 2-OHNa. ∑OHPh, sum of 1-OHPh, 2-OHPh, 3-OHPh, and 4-OHPh. ∑PAH, sum of all the 10 PAHs metabolites. | | |
|  | | |

| **Table S10** Sensitivity analysis for associations of urinary PAHs metabolites and urinary metals with lung function in NHANES 2011-2012 (excluding participants with asthma, *n*=962). | | | | | | | | | | | | | | |
| --- | --- | --- | --- | --- | --- | --- | --- | --- | --- | --- | --- | --- | --- | --- |
| Exposures | FVC, mL | |  | FEV_1_, mL | |  | FEV_1_/FVC (%) | |  | PEF, mL/s | |  | FEF _25-75%_, mL/s | |
|  | *β* (95% *CI*) | *P* |  | *β* (95% *CI*) | *P* |  | *β* (95% *CI*) | *P* |  | *β* (95% *CI*) | *P* |  | *β* (95% *CI*) | *P* |
| **Urinary PAHs** (ng/mmol creatinine) | | | | | | | | | | | | | | |
| 2-OHFlu | -63.11 (-181.63, 55.42) | 0.277 |  | -183.45 (-282.03, -84.86) | **0.001** |  | -3.24 (-4.66, -1.81) | **<0.001** |  | -563.56 (-968.87, -158.24) | **0.009** |  | -428.49 (-605.26, -251.73) | **<0.001** |
| 3-OHFlu | -26.46 (-118.00, 65.08) | 0.550 |  | -137.51 (-214.90, -60.11) | **0.002** |  | -2.85 (-4.04, -1.67) | **<0.001** |  | -464.81 (-806.34, -123.27) | **0.011** |  | -347.80 (-473.77, -221.83) | **<0.001** |
| 9-OHFlu | -160.96 (-319.06, -2.86) | **0.046** |  | -182.45 (-326.62, -38.28) | **0.016** |  | -1.33 (-2.76, 0.11) | 0.068 |  | -372.11 (-806.44, 62.22) | 0.088 |  | -238.68 (-518.83, 41.47) | 0.090 |
| 1-OHNa | 13.10 (-76.14, 102.34) | 0.761 |  | -58.44 (-132.50, 15.62) | 0.114 |  | -1.64 (-2.32, -0.96) | **<0.001** |  | -301.12 (-518.42, -83.82) | **0.010** |  | -207.84 (-323.95, -91.72) | **0.002** |
| 2-OHNa | 33.31 (-79.54, 146.15) | 0.542 |  | -116.78 (-228.09, -5.47) | **0.041** |  | -3.39 (-5.21, -1.57) | **0.001** |  | -431.13 (-826.52, -35.75) | **0.034** |  | -374.06 (-586.79, -161.32) | **0.002** |
| 1-OHPh | -107.16 (-233.76, 19.44) | 0.092 |  | -152.61 (-270.85, -34.37) | **0.015** |  | -1.03 (-2.68, 0.62) | 0.204 |  | -193.17 (-606.48, 220.13) | 0.338 |  | -219.00 (-432.34, -5.66) | **0.045** |
| 2-OHPh | -149.58 (-292.96, -6.21) | **0.042** |  | -200.98 (-338.63, -63.33) | **0.007** |  | -1.65 (-3.29, -0.004) | **0.050** |  | -367.92 (-834.95, 99.12) | 0.115 |  | -336.22 (-569.33, -103.11) | **0.007** |
| 3-OHPh | -77.43 (-188.33, 33.47) | 0.159 |  | -151.20 (-252.23, -50.16) | **0.006** |  | -1.97 (-3.41, -0.52) | **0.011** |  | -368.74 (-768.05, 30.57) | 0.068 |  | -308.65 (-508.40, -108.91) | **0.005** |
| 4-OHPh | -127.87 (-255.43, -0.31) | **0.050** |  | -160.88 (-280.14, -41.62) | **0.011** |  | -1.11 (-2.72, 0.50) | 0.165 |  | -103.69 (-504.28, 296.89) | 0.592 |  | -222.93 (-457.95, 12.09) | 0.062 |
| 1-OHP | -103.11 (-230.89, 24.67) | 0.107 |  | -175.39 (-291.55, -59.24) | **0.005** |  | -1.98 (-3.50, -0.45) | **0.014** |  | -361.10 (-743.38, 21.18) | 0.063 |  | -310.18 (-495.24, -125.13) | **0.003** |
| ∑OHFlu | -114.08 (-250.17, 22.01) | 0.095 |  | -204.10 (-325.46, -82.74) | **0.003** |  | -2.70 (-4.14, -1.25) | **0.001** |  | -526.68 (-957.05, -96.31) | **0.019** |  | -381.81 (-616.93, -146.70) | **0.003** |
| ∑OHNa | 25.75 (-103.84, 155.35) | 0.680 |  | -104.30 (-211.75, 3.14) | 0.056 |  | -2.91 (-4.06, -1.76) | **<0.001** |  | -441.56 (-740.83, -142.29) | **0.006** |  | -352.47 (-519.81, -185.12) | **<0.001** |
| ∑OHPh | -111.17 (-241.22, 18.89) | 0.089 |  | -170.48 (-289.83, -51.13) | **0.008** |  | -1.53 (-3.11, 0.05) | 0.058 |  | -271.24 (-689.29, 146.82) | 0.189 |  | -279.99 (-493.82, -66.17) | **0.013** |
| ∑PAH | 1.38 (-134.51, 137.27) | 0.983 |  | -126.26 (-238.52, -13.99) | **0.030** |  | -2.94 (-4.16, -1.73) | **<0.001** |  | -479.14 (-800.47, -157.82) | **0.006** |  | -370.65 (-552.45, -188.85) | **<0.001** |
| **Urinary metals** (ng/mmol creatinine) | | | | | | | | | | | | | | |
| Ba | -135.23 (-322.85, 52.40) | 0.147 |  | -96.28 (-245.33, 52.77) | 0.191 |  | 0.57 (-1.19, 2.32) | 0.505 |  | -150.40 (-512.09, 211.29) | 0.393 |  | -12.03 (-212.75, 188.69) | 0.901 |
| Cd | -57.82 (-203.86, 88.22) | 0.415 |  | -228.99 (-339.83, -118.15) | **<0.001** |  | -4.68 (-6.46, -2.91) | **<0.001** |  | -388.71 (-799.34, 21.92) | 0.062 |  | -502.89 (-727.71, -278.07) | **<0.001** |
| Co | -238.12 (-452.46, -23.78) | **0.032** |  | -181.03 (-377.27, 15.21) | 0.068 |  | 0.32 (-2.62, 3.26) | 0.821 |  | -245.81 (-959.21, 467.59) | 0.477 |  | -97.49 (-491.00, 296.01) | 0.608 |
| Cs | -60.95 (-300.48, 178.57) | 0.598 |  | -8.95 (-193.56, 175.66) | 0.920 |  | 1.20 (-1.35, 3.74) | 0.335 |  | 252.97 (-214.27, 720.21) | 0.269 |  | 61.59 (-341.71, 464.88) | 0.751 |
| Mo | -273.85 (-443.86, -103.84) | **0.003** |  | -110.55 (-277.99, 56.89) | 0.182 |  | 2.54 (0.75, 4.33) | **0.008** |  | 38.66 (-564.36, 641.69) | 0.894 |  | 158.21 (-195.60, 512.02) | 0.359 |
| Mn | -81.08 (-220.36, 58.20) | 0.236 |  | -25.73 (-156.80, 105.33) | 0.684 |  | 0.75 (-0.91, 2.41) | 0.354 |  | 349.84 (-147.69, 847.37) | 0.156 |  | 48.55 (-158.66, 255.75) | 0.627 |
| Pb | -12.65 (-229.23, 203.93) | 0.903 |  | -91.44 (-308.86, 125.99) | 0.387 |  | -2.13 (-3.84, -0.43) | **0.017** |  | -330.37 (-871.87, 211.14) | 0.215 |  | -222.74 (-596.17, 150.69) | 0.225 |
| Sb | -97.31 (-240.81, 46.19) | 0.171 |  | -55.55 (-168.05, 56.95) | 0.312 |  | 0.20 (-1.77, 2.16) | 0.836 |  | 59.15 (-355.87, 474.17) | 0.767 |  | 42.64 (-215.03, 300.31) | 0.731 |
| Tl | 74.16 (-175.91, 324.24) | 0.540 |  | 113.17 (-41.66, 268.01) | 0.141 |  | 2.18 (-0.98, 5.33) | 0.164 |  | 842.20 (218.88, 1465.52) | **0.011** |  | 291.13 (-113.10, 695.36) | 0.147 |
| W | -137.895 (-278.43, 2.66) | 0.054 |  | -76.70 (-244.28, 90.88) | 0.348 |  | 0.49 (-1.33, 2.31) | 0.579 |  | -232.28 (-641.26, 176.70) | 0.247 |  | -30.14 (-362.98, 302.71) | 0.851 |
| U | -125.58 (-283.32, 32.177) | 0.111 |  | -143.32 (-244.30, -42.34) | **0.008** |  | -1.20 (-3.33, 0.92) | 0.247 |  | -351.05 (-806.99, 104.89) | 0.123 |  | -106.92 (-329.76, 115.92) | 0.326 |
| **Note:** Log_10_-transformed, creatinine-corrected urinary concentration of each PAHs metabolite and metal were modeled as continuous variables to calculate coefficient and 95% confidence intervals (*CI*) for lung function. Generalized linear model adjusted for gender, age, race, height, family poverty-income ratio, smoking and drinking status. ∑OHFlu, sum of 2-OHFlu, 3-OHFlu, and 9-OHFlu. ∑OHNa, sum of 1-OHNa and 2-OHNa. ∑OHPh, sum of 1-OHPh, 2-OHPh, 3-OHPh, and 4-OHPh. ∑PAH, sum of all the 10 PAHs metabolites. | | | | | | | | | | | | | | |

| **Table S11** Sensitivity analysis for associations of urinary PAHs metabolites and urinary metals with lung function in NHANES 2011-2012 (excluding participants with COPD, *n*=1,087). | | | | | | | | | | | | | | |
| --- | --- | --- | --- | --- | --- | --- | --- | --- | --- | --- | --- | --- | --- | --- |
| Exposures | FVC, mL | |  | FEV_1_, mL | |  | FEV_1_/FVC (%) | |  | PEF, mL/s | |  | FEF _25-75%_, mL/s | |
|  | *β* (95% *CI*) | *P* |  | *β* (95% *CI*) | *P* |  | *β* (95% *CI*) | *P* |  | *β* (95% *CI*) | *P* |  | *β* (95% *CI*) | *P* |
| **Urinary PAHs** (ng/mmol creatinine) | | | | | | | | | | | | | | |
| 2-OHFlu | -44.57 (-170.50, 81.35) | 0.465 |  | -158.54 (-275.40, -41.68) | **0.011** |  | -3.21 (-5.08, -1.34) | **0.002** |  | -553.80 (-945.59, -162.02) | **0.008** |  | -342.09 (-544.78, -139.39) | **0.002** |
| 3-OHFlu | -20.70 (-119.48, 78.09) | 0.664 |  | -116.96 (-203.76, -30.16) | **0.011** |  | -2.68 (-4.26, -1.11) | **0.002** |  | -439.73 (-755.38, -124.07) | **0.009** |  | -268.93 (-413.40, -124.47) | **0.001** |
| 9-OHFlu | -134.18 (-288.07, 19.72) | 0.083 |  | -151.12 (-300.31, -1.93) | **0.047** |  | -1.24 (-3.36, 0.87) | 0.231 |  | -333.78 (-814.41, 146.86) | 0.161 |  | -162.03 (-462.64, 138.58) | 0.271 |
| 1-OHNa | 23.03 (-73.45, 119.51) | 0.621 |  | -47.50 (-140.23, 45.23) | 0.295 |  | -1.78 (-2.74, -0.81) | **0.001** |  | -318.49 (-563.91, -73.07) | **0.014** |  | -169.44 (-313.49, -25.38) | **0.024** |
| 2-OHNa | 40.01 (-51.52, 131.53) | 0.369 |  | -81.33 (-181.86, 19.21) | 0.106 |  | -2.82 (-4.73, -0.92) | **0.006** |  | -371.59 (-732.13, -11.05) | **0.044** |  | -234.26 (-452.76, -15.76) | **0.037** |
| 1-OHPh | -82.51 (-221.93, 56.91) | 0.287 |  | -115.85 (-238.47, 6.76) | 0.063 |  | -0.98 (-3.10, 1.15) | 0.347 |  | -134.28 (-517.49, 248.93) | 0.470 |  | -127.66 (-365.69, 110.37) | 0.274 |
| 2-OHPh | -132.96 (-280.15, 14.24) | 0.074 |  | -182.45 (-333.27, -31.63) | **0.021** |  | -1.81 (-3.90, 0.29) | 0.086 |  | -382.79 (-875.78, 110.19) | 0.120 |  | -273.24 (-510.22, -36.26) | **0.026** |
| 3-OHPh | -62.68 (-184.70, 59.33) | 0.294 |  | -136.81 (-257.17, -16.44) | **0.028** |  | -2.19 (-4.22, -0.15) | **0.037** |  | -407.91 (-801.78, -14.05) | **0.043** |  | -250.44 (-474.73, -26.15) | **0.031** |
| 4-OHPh | -102.66 (-226.16, 20.84) | 0.098 |  | -154.96 (-284.88, -25.04) | **0.022** |  | -1.66 (-4.07, 0.75) | 0.165 |  | -140.11 (-566.65, 286.43) | 0.498 |  | -213.45 (-465.45, 38.56) | 0.092 |
| 1-OHP | -71.76 (-208.81, 65.30) | 0.285 |  | -132.57 (-257.42, -7.72) | **0.039** |  | -1.83 (-3.74, 0.09) | 0.060 |  | -316.29 (-681.42, 48.83) | 0.085 |  | -213.95 (-410.13, -17.78) | **0.034** |
| ∑OHFlu | -93.04 (-233.75, 47.67) | 0.181 |  | -175.21 (-308.68, -41.74) | **0.013** |  | -2.62 (-4.67, -0.57) | **0.015** |  | -502.59 (-936.75, -68.44) | **0.026** |  | -293.96 (-554.84, -33.07) | **0.029** |
| ∑OHNa | 35.12 (-86.77, 157.02) | 0.551 |  | -76.67 (-191.79, 38.45) | 0.178 |  | -2.63 (-3.93, -1.32) | **<0.001** |  | -409.47 (-709.89, -109.05) | **0.011** |  | -244.08 (-436.32, -51.84) | **0.016** |
| ∑OHPh | -86.41 (-226.38, 53.56) | 0.210 |  | -142.88 (-275.79, -9.97) | **0.037** |  | -1.70 (4.00, 0.61) | 0.139 |  | -261.36 (-671.97, 149.26) | 0.197 |  | -204.09 (-454.24, 46.06) | 0.103 |
| ∑PAH | 14.37 (-117.35, 146.08) | 0.821 |  | -95.73 (-219.06, 27.60) | 0.120 |  | -2.68 (-4.13, -1.23) | **0.001** |  | -444.57 (-773.72, -115.42) | **0.011** |  | -257.69 (-467.49, -47.90) | **0.019** |
| **Urinary metals** (ng/mmol creatinine) | | | | | | | | | | | | | | |
| Ba | -99.86 (-269.85, 70.13) | 0.232 |  | -69.16 (-211.39, 73.07) | 0.319 |  | 0.53 (-1.18, 2.24) | 0.520 |  | -128.19 (-544.19, 287.82) | 0.524 |  | -0.62 (-202.81, 201.57) | 0.995 |
| Cd | -16.12 (-148.93, 116.69) | 0.801 |  | -148.04 (-256.11, -39.97) | **0.010** |  | -3.63 (-5.46, -1.80) | **<0.001** |  | -298.68 (-673.38, 76.02) | 0.111 |  | -342.19 (-568.12, -116.26) | **0.005** |
| Co | -187.49 (-401.40, 26.42) | 0.082 |  | -166.71 (-333.98, 0.571) | 0.051 |  | -0.31 (-2.94, 2.33) | 0.810 |  | -269.07 (-903.88, 365.73) | 0.384 |  | -123.65 (-482.58, 235.27) | 0.477 |
| Cs | -22.40 (-235.72, 190.92) | 0.827 |  | 11.40 (-167.36, 190.15) | 0.895 |  | 0.54 (-1.49, 2.56) | 0.585 |  | 250.88 (-205.77, 707.52) | 0.262 |  | 83.12 (-252.35, 418.60) | 0.608 |
| Mo | -238.93 (-394.39, -83.46) | **0.005** |  | -79.31 (-241.44, 82.82) | 0.317 |  | 2.51 (-0.03, 5.04) | 0.052 |  | -22.94 (-575.73, 529.85) | 0.931 |  | 162.21 (-210.52, 534.93) | 0.371 |
| Mn | -50.63 (-185.19, 83.94) | 0.438 |  | -19.01 (-131.18, 93.16) | 0.725 |  | 0.19 (-1.65, 2.02) | 0.831 |  | 232.59 (-257.95, 723.14) | 0.331 |  | 33.73 (-135.71, 203.17) | 0.680 |
| Pb | 57.75 (-156.94, 272.44) | 0.578 |  | -15.85 (-226.24, 194.54) | 0.876 |  | -1.61 (-3.22, 0.0005) | 0.050 |  | -223.11 (-725.75, 279.53) | 0.362 |  | -132.86 (-471.01, 205.29) | 0.419 |
| Sb | -66.39 (-207.32, 74.55) | 0.334 |  | -29.28 (-129.57, 71.01) | 0.546 |  | 0.04 (-1.40, 1.48) | 0.958 |  | 94.38 (-284.28, 473.05) | 0.606 |  | 81.28 (-126.74, 289.30) | 0.421 |
| Tl | 43.16 (-172.13, 258.45) | 0.678 |  | 102.70 (-69.58, 274.98) | 0.226 |  | 2.18 (-0.49, 4.85) | 0.103 |  | 795.86 (245.19, 1,346.54) | **0.007** |  | 303.90 (-63.52, 671.31) | 0.099 |
| W | -119.00 (-262.25, 24.25) | 0.098 |  | -58.38 (-202.13, 85.38) | 0.404 |  | 0.44 (-1.11, 2.00) | 0.555 |  | -206.99 (-554.66, 140.68) | 0.226 |  | 14.17 (-255.95, 284.28) | 0.913 |
| U | -131.51 (-315.42, 52.40) | 0.150 |  | -153.29 (-265.47, -41.11) | **0.010** |  | -1.37 (-3.57, 0.82) | 0.204 |  | -356.01 (-751.79, 39.78) | 0.075 |  | -111.01 (-318.21, 96.20) | 0.274 |
| **Note:** Log_10_-transformed, creatinine-corrected urinary concentration of each PAHs metabolite and metal were modeled as continuous variables to calculate coefficient and 95% confidence intervals (*CI*) for lung function. Generalized linear model adjusted for gender, age, race, height, family poverty-income ratio, smoking and drinking status. ∑OHFlu, sum of 2-OHFlu, 3-OHFlu, and 9-OHFlu. ∑OHNa, sum of 1-OHNa and 2-OHNa. ∑OHPh, sum of 1-OHPh, 2-OHPh, 3-OHPh, and 4-OHPh. ∑PAH, sum of all the 10 PAHs metabolites. | | | | | | | | | | | | | | |

| **Table S12** Comparisons of four statistical methods. | | | | | |
| --- | --- | --- | --- | --- | --- |
| Methods | Description |  | Advantages |  | Limitations |
| GLM | GLM theory is predicated on the exponential family of distributions, and it includes the commonly used normal, binomial, gamma, and Poisson distributions. |  | Extensively employed due to their simplicity and ease to explain, GLM is used to ascertain the associations between exposures and human health. |  | Misleading conclusions may be drawn in the cases of multiple comparisons, multi-collinearity, and high dimensionality. |
| LASSO | LASSO penalized regression analysis is used to select significant variables associated with outcomes through the construction of penalty function. This approach not only simplifies the model’s complexity but also avoids the over-fitting problem caused by the correlation between variables. |  | A robust method for addressing multi-collinearity, reducing dimensionality, and identifying significant components. |  | Only fits a linear model, and only one component is chosen from a group of highly correlated pollutants and lacks results that provide information on the effect size, such as the percent change obtained in the other approaches. |
| BKMR | BKMR uses a Gaussian kernel function to flexibly evaluate exposure-response functions, thereby enabling the identification of non-linear and non-additive associations, as well as interactions of multiple variables. |  | Addressing the problems of nonlinearity, interactive effects as well as overall effects and component selection, which could further capture the association between multiple variables and outcomes. |  | The kernel algorithm that fixes other exposures at a certain level limits the estimation of co-exposure patterns involving both high and low levels of chemicals. |
| Qgcomp | Qgcomp classifies all variables into quartiles and uses these quantified exposures as continuous variables to fit a linear regression model. Mixing effect is assessed by considering the simultaneous increase in all variables by one quartile. Each variable was assigned to a positive or negative weight, reflecting its individual contribution to the overall effect. |  | Allowing unbiased inference of multi-pollutant mixing effect and identifying significant contributors to outcomes. |  | Data information may be lost during transformation to quantiles. |
| **Abbreviations:** BKMR, Bayesian kernel machine regression; GLM, generalized linear model; LASSO, least absolute shrinkage and selection operator; Qgcomp, quantile-based g-computation. | | | | | |





**Figure S1** The flowchart of study design.

**Abbreviations:** PIR, poverty-income ratio; PAHs, polycyclic aromatic hydrocarbons.


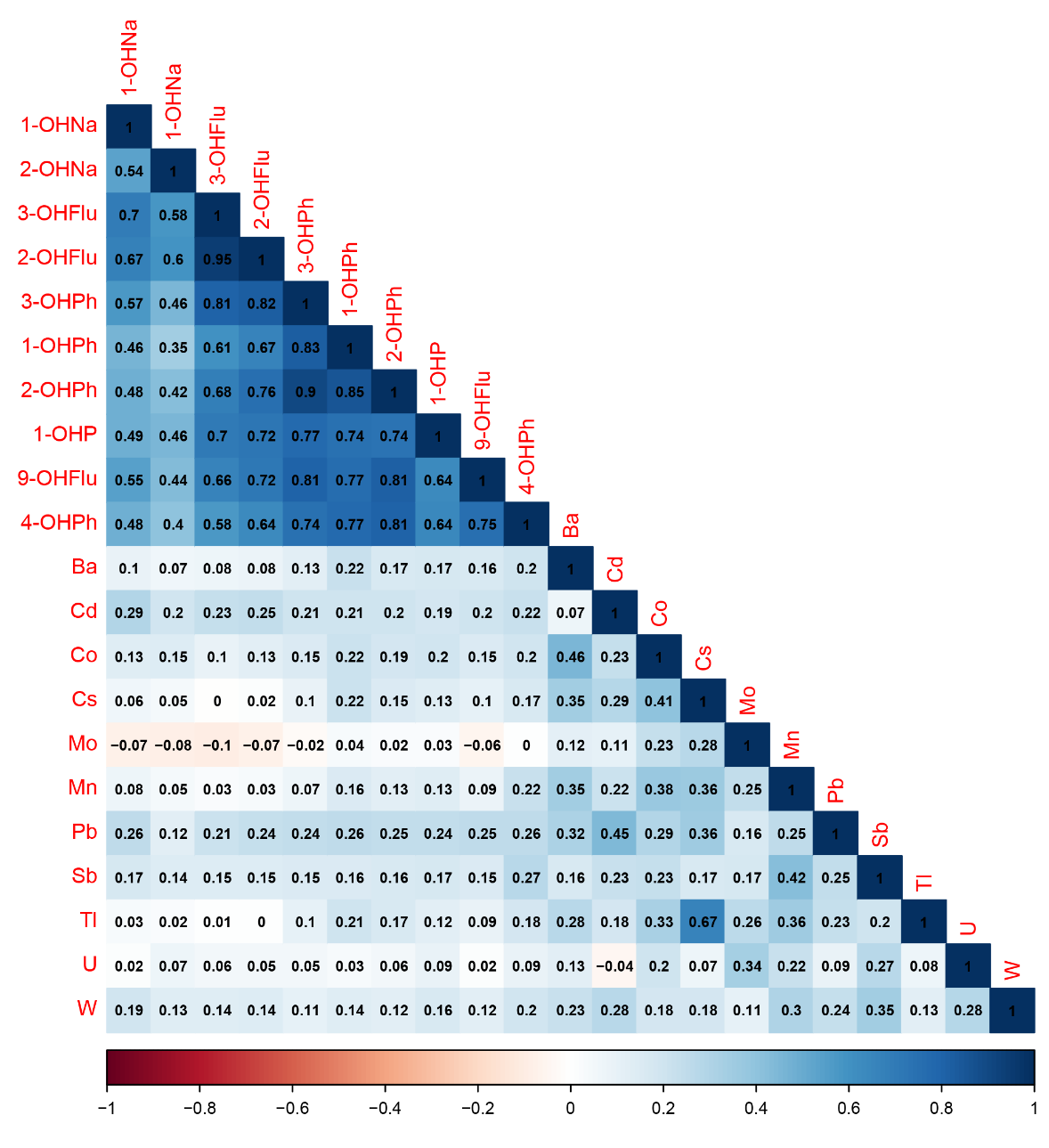


**Figure S2** Pearson correlations between urinary concentrations of 10 PAH metabolites and 11 metals.

**Note:** All the correlations were statistically significant (all *P* <0.001).


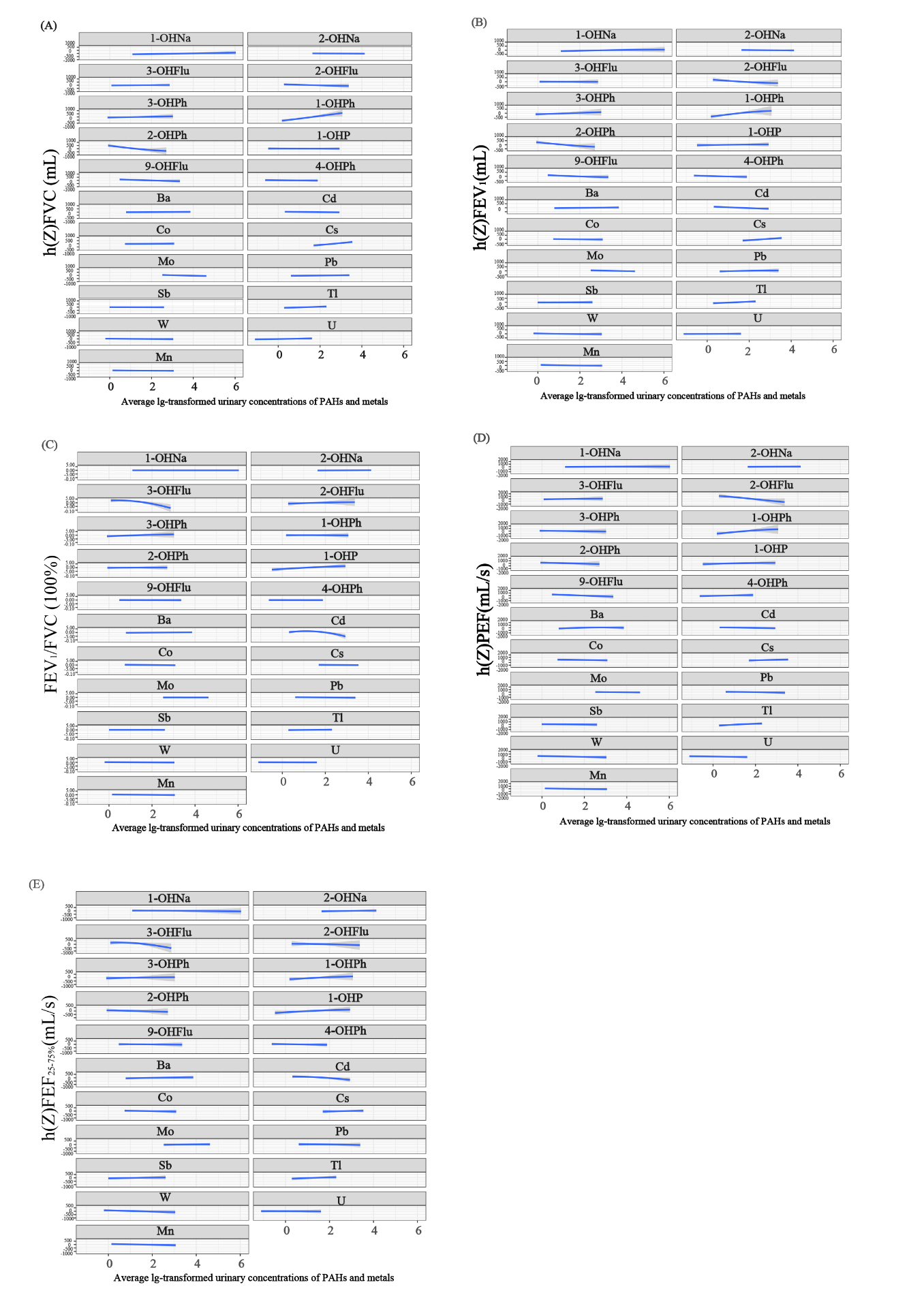
**Figure S3** Univariate exposure-response functions and 95% *CI* for each urinary PAHs and metals.


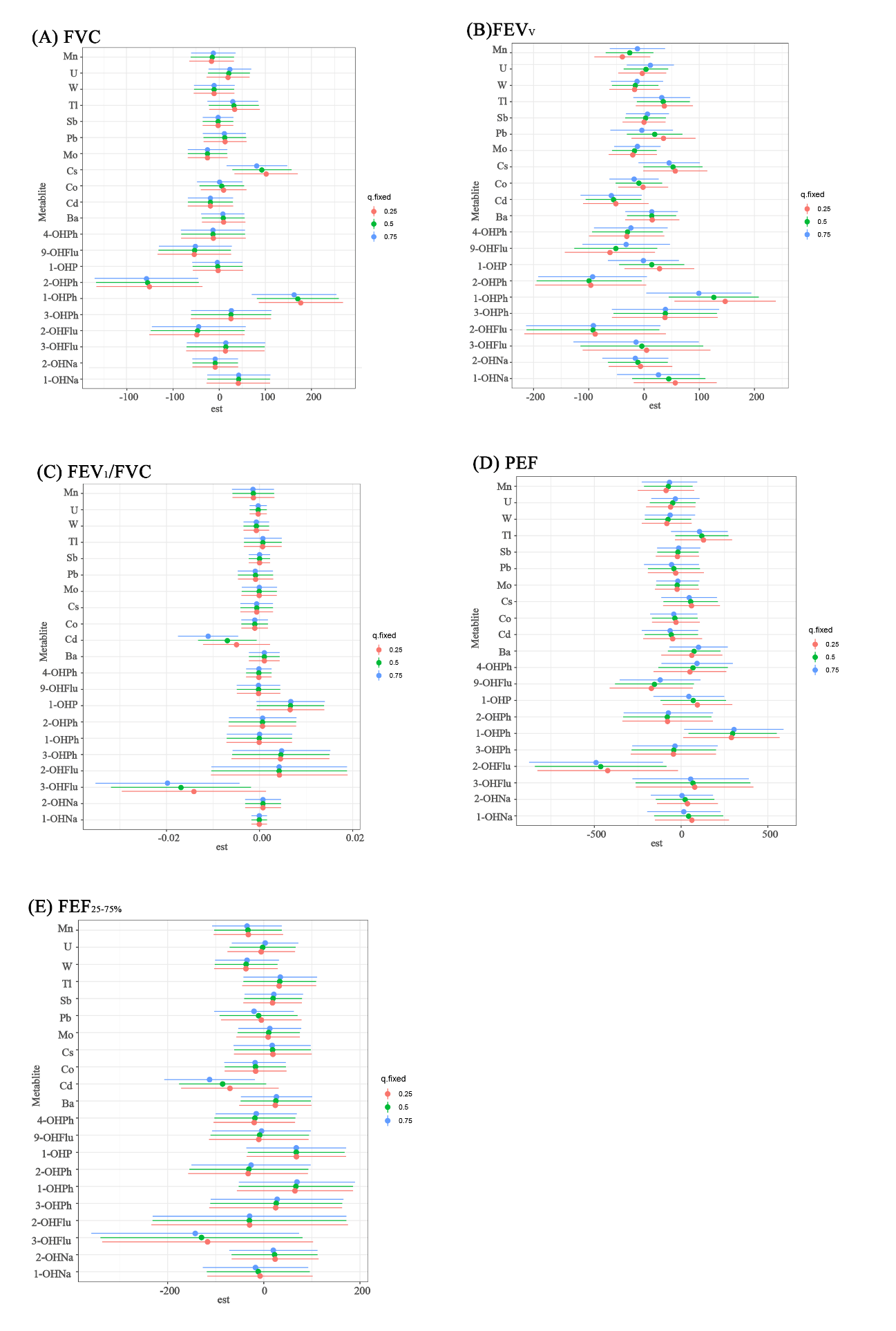
**Figure S4** The single-exposure effect (estimates and 95% *CI*) for urinary PAHs and metals and lung function.


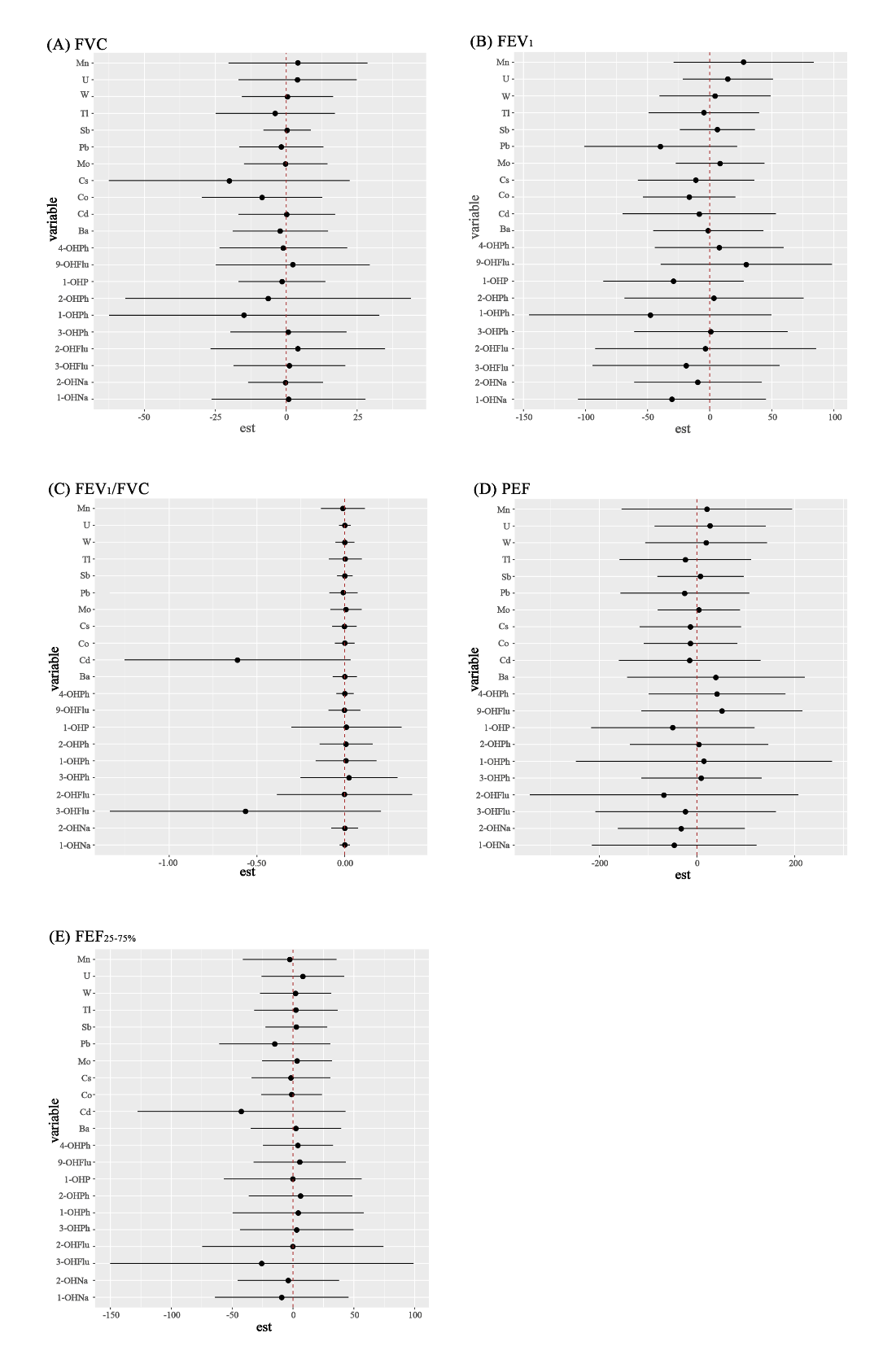


**Figure S5** Single variable interaction summary for urinary PAHs and metals on lung function.


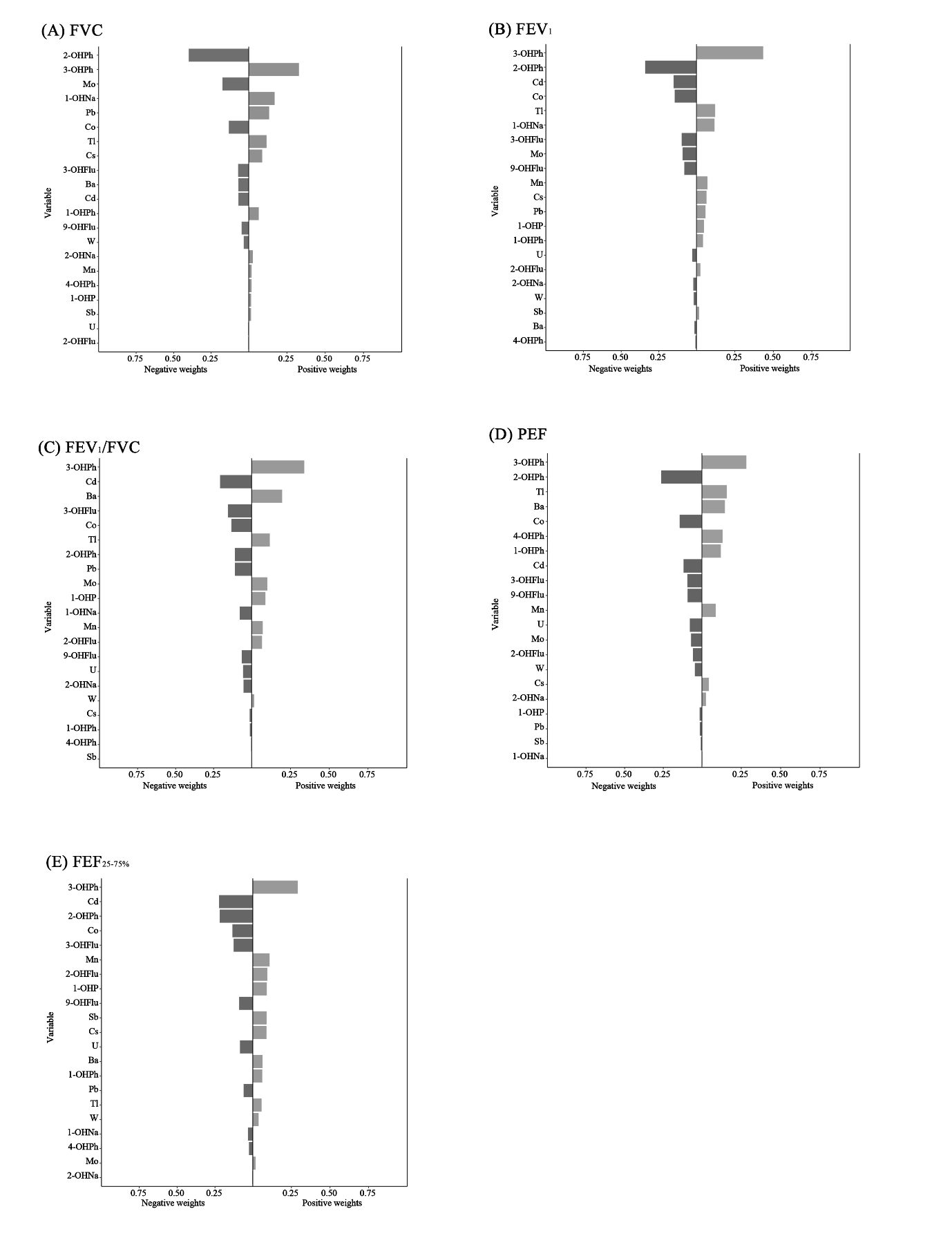


**Figure S6** Weight of each PAHs and metals in the association with lung function by quantile-based g-computation regression.
