# Supplementary material for: Co-exposure effects of urinary polycyclic aromatic hydrocarbons and metals on lung function: mediating role of systematic inflammation
Source: BMC Pulm Med. 2024 Aug 11;24:386. doi: 10.1186/s12890-024-03173-9 (PMC11316979; doi:10.1186/s12890-024-03173-9)
Supplement: Supplementary file 2 — Supplementary Material 2. [file 12890_2024_3173_MOESM2_ESM.docx]

RIAGENDR - Gender

**English Text:**

Gender of the participant.

| **Code or Value** | **Value Description** |
| --- | --- |
| 1 | Male |
| 2 | Female |
| . | Missing |

RIDAGEYR - Age in years at screening

**English Text:**

Age in years of the participant at the time of screening. Individuals 80 and over are topcoded at 80 years of age.

| **Code or Value** | **Value Description** |
| --- | --- |
| 0 to 79 | Range of Values |
| 80 | 80 years of age and over |
| . | Missing |

RIDRETH1 - Race/Hispanic origin

**English Text:**

Recode of reported race and Hispanic origin information

| **Code or Value** | **Value Description** |
| --- | --- |
| 1 | Mexican American |
| 2 | Other Hispanic |
| 3 | Non-Hispanic White |
| 4 | Non-Hispanic Black |
| 5 | Other Race - Including Multi-Racial |
| . | Missing |

INDFMPIR - Ratio of family income to poverty

**English Text:**

A ratio of family income to poverty guidelines.

| **Code or Value** | **Value Description** |
| --- | --- |
| 0 to 4.99 | Range of Values |
| 5 | Value greater than or equal to 5.00 |
| . | Missing |

SMQ020 - Smoked at least 100 cigarettes in life

**English Text:**

These next questions are about cigarette smoking and other tobacco use. {Have you/Has SP} smoked at least 100 cigarettes in {your/his/her} entire life?

| **Code or Value** | **Value Description** |
| --- | --- |
| 1 | Yes |
| 2 | No |
| 7 | Refused |
| 9 | Don't know |
| . | Missing |

ALQ101 - Had at least 12 alcohol drinks/1 yr?

**English Text:**

The next questions are about drinking alcoholic beverages. Included are liquor (such as whiskey or gin), beer, wine, wine coolers, and any other type of alcoholic beverage.In any one year, {have you/has SP} had at least 12 drinks of any type of alcoholic beverage? By a drink, I mean a 12 oz. beer, a 5 oz. glass of wine, or one and half ounces of liquor.

| **Code or Value** | **Value Description** |
| --- | --- |
| 1 | Yes |
| 2 | No |
| 7 | Refused |
| 9 | Don't know |
| . | Missing |
